# Nanoscale Surface and Bulk Electronic Properties of Ti_3_C_2_T_x_ MXene Unraveled by Multimodal X‐Ray Spectromicroscopy

**DOI:** 10.1002/smtd.202400190

**Published:** 2024-06-14

**Authors:** Faidra Amargianou, Peer Bärmann, Hui Shao, Pierre‐Louis Taberna, Patrice Simon, Jesus Gonzalez‐Julian, Markus Weigand, Tristan Petit

**Affiliations:** ^1^ Helmholtz‐Zentrum Berlin für Materialien und Energie GmbH Albert‐Einstein‐Straße 15 12489 Berlin Germany; ^2^ Faculty of Mathematics and Natural Sciences TU‐Berlin Hardenbergstr. 36 10623 Berlin Germany; ^3^ Université Paul Sabatier CIRIMAT UMR CNRS 5085, 118 route de Narbonne Toulouse 31062 France; ^4^ Institute of Mineral Engineering (GHI) Chair of Ceramics RWTH Aachen 52074 Aachen Germany

**Keywords:** 2D layered material, Li‐ion battery, MXenes, X‐ray microscopy

## Abstract

2D layered materials, such as transition metal carbides or nitrides, known as MXenes, offer an ideal platform to investigate charge transfer processes in confined environment, relevant for energy conversion and storage applications. Their rich surface chemistry plays an essential role in the pseudocapacitive behavior of MXenes. However, the local distribution of surface functional groups over single flakes and within few‐ or multilayered flakes remains unclear. In this work, scanning X‐ray microscopy (SXM) is introduced with simultaneous transmission and electron yield detection, enabling multimodal nanoscale chemical imaging with bulk and surface sensitivity, respectively, of individual MXene flakes. The Ti chemical bonding environment is found to significantly vary between few‐layered hydrofluoric acid‐etched Ti_3_C_2_T_x_ MXenes and multilayered molten salt (MS)‐etched Ti_3_C_2_T_x_ MXenes. Postmortem analysis of MS‐etched Ti_3_C_2_T_x_ electrodes cycled in a Li‐ion battery further illustrates that simultaneous bulk and surface chemical imaging using SXM offers a method well adapted to the characterization of the electrode‐electrolyte interactions at the nanoscale.

## Graphical abstract

Multimodal scanning X‐ray microscopy with surface and bulk sensitivity, enabled by the simultaneous detection of emitted electrons and transmitted X‐rays, is introduced for the chemical imaging of 2D‐layered materials. This method allows the characterization of the local chemistry of individual Ti_3_C_2_T*_x_* MXene flakes produced by different synthesis routes and after cycling in a Li‐ion battery.

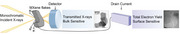

## 1 Introduction

Van der Waals interactions in 2D layered materials play a pivotal role in the physical properties of the individual 2D sheets. The topmost layer may have other properties than buried layers, which are strongly influenced by Van der Waals forces depending on interlayer distances.^[^ ^1^, ^2^, ^3^ ^]^ The introduction of guest species through intercalation offers a means to fine‐tune the chemical and electronic interactions between these layers,^[^ ^4^, ^5^ ^]^ which can have profound implications for electrochemical energy storage applications.^[^ ^6^, ^7^ ^]^ While the surface layer may exhibit unique electronic and chemical properties in contrast to the buried layers for few‐layered materials, a comprehensive study for multilayered materials remains elusive due to a scarcity of probing techniques offering both bulk and surface sensitivity down to single layers.

2D layered transition metal carbides, carbonitrides and nitrides, known as MXenes have a versatile chemistry that allows the tuning of properties for applications including energy storage,^[^ ^8^ ^]^ optoelectronics,^[^ ^9^, ^10^ ^]^ electromagnetic interference shielding,^[^ ^11^ ^]^ water purification,^[^ ^12^ ^]^ and gas sensors.^[^ ^13^ ^]^ Briefly, MXenes are 2D materials with the formula of M_n+1_X_n_T_x_, (*n* = 1–4), where M represents an early transition metal(s), X is either carbon and/or nitrogen, and T_x_ corresponds to the terminal groups (─F, ─OH, ─O, etc.) formed during the etching process. By now, a great number of MXene compositions have been discovered, but Ti_3_C_2_T_x_ MXene remains the most studied one, thanks to its easy synthesis, good stability, and excellent electrochemical properties.

MXenes have shown promising properties for Li‐ion batteries^[^ ^14^, ^15^ ^]^ and supercapacitors.^[^ ^16^ ^]^ Yet, fundamental questions persist about its pseudocapacitive energy storage mechanisms. Surface/confined redox reactions in MXene flake, potentially influenced by Solid‐Electrolyte Interphase (SEI) formation, might exhibit distinct characteristics compared to those within its interlayer spaces, emphasizing the need for thorough investigations of both surface and bulk chemical bonding.^[^ ^17^ ^]^ Most of the MXene currently applied in electrochemical energy storage devices are based on wet chemical etching of a MAX phase with hydrofluoric acid (HF)–containing solutions (HF‐Ti_3_C_2_T_x_),^[^ ^18^ ^]^ or Lewis acid etching using molten salts (MS‐Ti_3_C_2_T_x_).^[^ ^19^ ^]^ Ti_3_C_2_T_x_ with F‐rich terminating species are expected to be highly resilient to oxidation.^[^ ^20^ ^]^ On the other hand, MS‐Ti_3_C_2_T_x_ exhibited excellent electrochemical performance in Li‐ion‐contained non‐aqueous electrolytes compared to HF‐Ti_3_C_2_T_x_.^[^ ^19^ ^]^ Individual Ti_3_C_2_T_x_ MXene sheets are generally stacked to form few‐layered or multi‐layered flakes depending on the synthesis and delamination process.

Capturing the surface and bulk properties of monolayer, few‐layered, and multi‐layered MXene flakes remains experimentally very challenging. While electron loss spectroscopy has enabled a great understanding of the structure of single MXene flakes, it does not allow high energy resolution to capture the fine Ti chemical bonding.^[^ ^21^ ^]^ Similarly, scanning probe techniques such as tip‐enhanced Raman spectroscopy allow high spatial resolution but are limited to the top MXene layer of a flake.^[^ ^22^ ^]^ Additionally, layered transition metal compounds stand out due to the potent electronic correlations of the transition metal *d* orbitals and their high sensitivity to changes in the surrounding chemical environment.^[^ ^23^ ^]^ There is a strong need for a non‐destructive technique that will allow probing both the surface and bulk of MXene multilayered flakes with a large chemical sensitivity to the transition metal chemical bonding.

Soft X‐ray absorption spectroscopy (XAS) allows the probing of the fine electronic structure of the metal valence electrons and light elements present in the layered transition metal compounds and electrolytes.^[^ ^24^, ^25^ ^]^ Specifically, for transition metal structures with termination groups found in MXenes, the crystal field interaction determines the shape, strength, and occupancy of electronic orbitals.^[^ ^26^ ^]^ X‐ray PhotoElectron Emission Microscopy (XPEEM) has already been employed for investigating modification of the MXene surface chemistry upon cation intercalation in different individual Ti_3_C_2_T_x_ flakes with sub‐50 nanometer spatial resolution.^[^ ^27^, ^28^ ^]^ However, XPEEM is a surface‐sensitive technique due to the detection of photoemitted electrons only. While XPEEM has been previously performed on Li‐ion battery electrodes, this approach is challenging due to the morphological complexity of such materials, characterized by very porous and rough surfaces.^[^ ^29^ ^]^

Transmission imaging techniques with soft X‐rays are ideal for monitoring changes in the local chemistry of few‐layered materials, with guest species between the interlayers or exposed to different environments. Full‐field transmission X‐ray microscopy (TXM) is capable of 2D imaging and tomography, as well as spectroscopic imaging. It has been employed as a useful tool to study morphology, chemical distributions, reaction dynamics, and degradation mechanisms in 2D layered and electrode materials.^[^ ^30^ ^]^ However, the rigid configuration of TXM is not appropriate for the parallel collection of complementary information using a multimodal approach.^[^ ^31^ ^]^ Unlike TXM, Scanning Transmission X‐ray Microscopy (STXM) offers flexibility in adjusting the field of view. In addition to transmission detection, total electron yield (TEY) detection^[^ ^32^ ^]^ has already been demonstrated, which makes this technique ideal for a multimodal characterization of both surface and bulk chemistry.

In this work, we apply multimodal Scanning X‐ray Microscopy (SXM) for simultaneous hyperspectral imaging of bulk and surface electronic properties of individual Ti_3_C_2_T_x_ MXene flakes. Single and few‐layered HF‐etched Ti_3_C_2_T_x_ MXenes and multilayered MS‐etched Ti_3_C_2_T_x_ MXenes are imaged by multimodal SXM. Transmission detection mode is used for bulk sensitivity, required to probe the surface chemistry in the MXene interlayer, while TEY mode allows high surface sensitivity for probing the top MXene layer. The results unravel the influence of different processing routes on Ti chemical bonding monitored by XAS at the Ti L‐ and O K‐edge. Multimodal SXM enables the chemical identification of the different components found in postmortem analysis of MS‐etched Ti_3_C_2_T_x_ MXenes cycled in a Li‐ion battery. Adsorbed electrolyte (surface) and electrolyte‐electrode interaction (bulk) can be probed with high chemical sensitivity. Changes in the chemical bonding configuration of cycled MXene electrode are recorded in the bulk of MXene, related to the interaction of MXene with intercalated lithium cations, and its top layer, due to interaction with the electrolyte. This approach can contribute to deciphering intercalation from surface redox reactions in other laminated materials.

## 2 Methods

### 2.1 Simultaneous Surface‐ and Bulk‐Sensitive X‐Ray Spectromicroscopy

**Figure 1:**
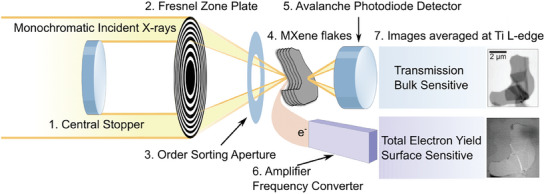
Scheme of Scanning X‐ray Microscopy (SXM) Setup. Central stopper (1) eliminates non‐diffracted (0th order) monochromatic X‐rays coming from the synchrotron light source. A FZP (2) focuses the X‐ray light and an OSA (3) allows only 1st‐order diffracted X‐rays impinging on a spot of the MXene flakes (4). The MXene samples are raster‐scanned. The X‐rays, transmitted through the MXene flakes, are recorded by an avalanche photodiode detector (5). The drain current from the MXene sample, resulting from the electrons emission from MXene's top layer, is converted to frequencies by the amplifier frequency converter (6). The SXM images are recorded on Ti_3_C_2_T_x_ MXene in both transmission and TEY modes and an example of an SXM image averaged over the Ti L‐edge energy range is shown (7).

In a conventional SXM setup, monochromatic X‐rays are demagnified through a Fresnel Zone Plate (FZP), generating a finely focused microprobe. This microprobe scans across the specimen under examination in a raster pattern. An Order‐Selecting Aperture (OSA) is placed between the FZP and the specimen to filter out all undesired diffraction orders, as shown in **Figure** **1**. Multimodal SXM allows the detection of both transmitted X‐rays (transmission mode) and drain current of released photoelectrons (TEY mode).^[^ ^32^ ^]^ The bulk‐sensitive transmission mode is acquired with an avalanche photodiode detector (APD) while the surface‐sensitive TEY mode is recorded by amplifying the sample drain current. A bias voltage of +30 V is set between the sample holder and the OSA to remove parasitic electrons.

Due to the short penetration depth of soft X‐rays, thin samples are required for transmission detection,^[^ ^32^ ^]^ which may be challenging for the characterization of multilayered MXenes. While a single Ti_3_C_2_T_x_ MXene layer is ≈1 nm in thickness, multilayered MXene typically comprises stacked MXene layers of micrometric thickness that are too thick for transmission detection. Delaminated Ti_3_C_2_T_x_ MXene, dispersed in water, on the other hand, primarily comprises single and few‐layered MXene flakes with lateral dimensions of a few micrometers that can be used to form very thin films. With transmission measurements, saturation can occur when either the sample thickness or the absorption coefficient µ is too large.^[^ ^33^ ^]^ For the characterization of Ti_3_C_2_T_x_ MXene at the Ti L‐edge, we estimate the thickness limit to ≈40 MXene layers.

In contrast, the surface‐sensitive TEY mode is not limited by the sample thickness and allows X‐ray microscopic examination of samples too thick for conventional transmission detection. At the Ti L‐edge, the information depth is ≈2–4 nm, therefore mostly probing the first 1–2 MXene layers, regardless of the flake thickness. More details on the information and probing depths of both transmission and TEY are available in the Supporting Information.

### 2.2 Hyperspectral X‐Ray Imaging

**Figure 2:**
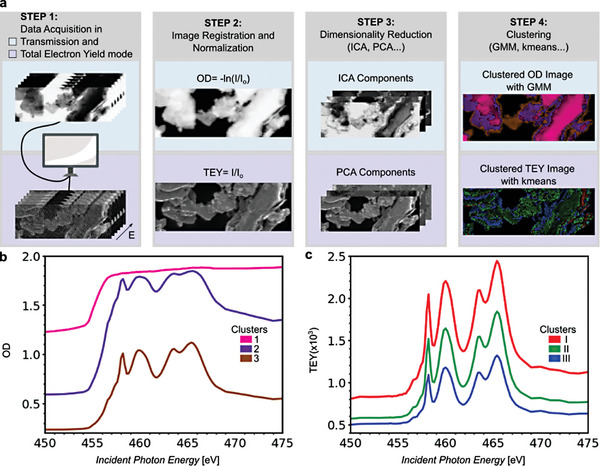
a) Workflow for spectromicroscopic data, collected for multilayered MS‐Ti_3_C_2_T_x_ MXene. Images in transmission and TEY mode, acquired at Ti L‐edge (step 1) are preprocessed for drift correction and normalization (step 2). Then, images are reduced to a few components by performing a dimensionality reduction (step 3). Clustering is performed on these components to create the clustered transmission and TEY image (step 4). Clustered spectrum is extracted by averaging the preprocessed spectra, corresponding to pixels of a clustered area. Clustered spectra are shown in (b) for transmission and (c) for TEY mode. Clusters 1–3 and clusters I–III are produced after clustering separately the preprocessed transmission and TEY data, respectively. Scalebar: 2 µm.

Multimodal SXM enables hyperspectral imaging where XAS is acquired simultaneously in transmission and TEY detection mode for each N‐dimensional pixel, where N is the number of different energy values. A workflow for the analysis of spectromicroscopic data collected for multilayered Ti_3_C_2_T_x_ MXene is presented in **Figure** **2**. The process begins with the acquisition of images in both transmission and TEY modes at the Ti L‐edge (step 1) with a typical pixel size of 50 nm.

Image registration with cross‐correlation is performed to the stack of transmission and TEY images to correct the drift during scanning. Transmission data are converted to optical density (OD) and TEY data are divided by I_o_ (step 2). Subsequently, dimensionality reduction techniques, such as Independent Component Analysis (ICA) and Principal Component Analysis (PCA) are applied to the images, reducing them to a few essential components (step 3). Clustering using models, such as the Gaussian Mixture Model (GMM) and k‐means, is performed on these components to create the clustered OD and TEY images (step 4). The suitable number of components and clusters is estimated by the elbow and silhouette method (Figure S3, Supporting Information), together with the criteria for the selection of dimensionality reduction and clustering techniques. From these clustered images, the corresponding X‐ray absorption (XA) spectra are extracted by averaging the preprocessed spectra for pixels within a given clustered area. The clustered XA spectra are represented in both transmission (Figure 2b) and TEY modes (Figure 2c). The clustered spectra in transmission mode correspond mainly to areas with different thicknesses, whereas the ones in TEY, are by areas with different electron emission behavior.

The main challenge is clustering the pixels that present similar chemical information independently from the MXene thickness or electron emission properties. In Figure 2c, clusters 1 and 2 correspond to thicker areas with transmission spectra presenting absorption saturation distortions. Cluster 3 corresponds to the thinner part of the MXene sample, yielding a transmission XAS Ti L‐edge spectrum with distinct peaks that will be discussed in the following. Clusters I, II, and III obtained from the TEY mode correspond to MXene areas with decreasing electron emission. Cluster I corresponds to areas with increased TEY signal, caused by edge enhancement, given that these areas are usually the edges of the MXene flakes. On the other hand, a decreased signal of Cluster III can be explained by the charging of the sample.

## 3 Results

### 3.1 Multimodal SXM Imaging of Individual Ti_3_C_2_T_x_ MXene Flakes

**Figure 3:**
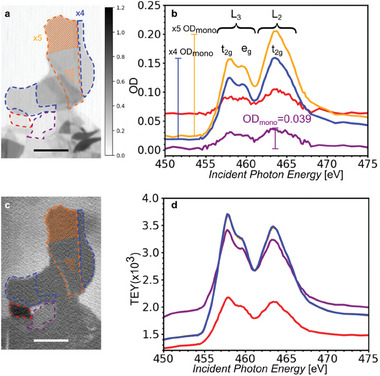
a) Transmission image, averaged at Ti L‐edge, highlighting regions corresponding to a monolayer (dotted violet), 4‐layered (dotted blue), and 5‐layered HF‐Ti_3_C_2_T_x_ MXene flakes, with corresponding transmission XA spectra extracted from the orange dashed area (b). A monolayer MXene flake with reduced electron yield (dotted red) is also indicated. c) TEY image at 450 eV depicting the same regions as in a, with corresponding TEY XA spectra (d). Scalebar: 2 µm.

Representative SXM images of few‐layered Ti_3_C_2_T_x_ MXenes at the Ti L‐edge are shown in **Figure** **3**. Overlapping single and few‐layered flakes are visible in transmission and TEY mode. The comparison of transmission and TEY images reveals critical insights. The variation in OD in transmission images provides a detailed view of the thickness of MXene flakes, down to a monolayer (Figure 3a). The transmission XA spectra at the Ti L‐edge have similar spectral signatures throughout the flake (Figure 3b). XAS at Ti L‐edge is induced by the excitation of Ti 2p electron to the unfilled 3d orbitals. Ti L_3_ and L_2_ edges arise from Ti 2p_3/2_ to Ti 3d and Ti 2p_1/2_ to Ti 3d transitions, respectively.^[^ ^34^ ^]^ XAS can be employed for analysis of average *t*_2g_ *–e*_g_ crystal field splitting and relative *t*_2g_ to *e*_g_ bands occupancy^[^ ^35^ ^]^ as discussed in the following section. In Figure 3a,b the main differences in spectra between the MXene areas are related to the thickness.

OD_mono_ is defined as the maximum L_3_ peak intensity for a monolayer flake and is estimated to be 0.039 ± 0.005 (Figure S5, Supporting Information). Given the maximum OD of a MXene flake, we can predict the number of layers of a flake, by exploiting the linear relation of OD with thickness. Specifically, a 4‐layered flake (blue dotted), with several flakes lying on top, is identified in Figure 3a, based on its respective maximum OD in the transmission spectrum (Figure 3b). It is partially covered by other flakes, except for the dashed orange areas corresponding to 5 layers, whose spectrum is provided. Therefore, we can state that a monolayer (dotted orange) lays on top of a 4‐layered flake (dotted orange).

The surface sensitivity of TEY leads to a relatively uniform imaging of the MXene sample (Figure 3c). The blue‐colored spectrum is identical to the orange‐colored one (Figure 3d). Some other areas have also similar TEY XA spectra but shifted to higher or lower TEY. This difference is irrelevant to the thickness and is rather related to various TEY phenomena, as discussed in  Supporting Information and in Figures S6–S8 (Supporting Information).

TEY images are utilized to identify areas with different electron emission properties, such as the MXene flake shown in dotted red (Figure 3c). Figure 3a,b presents indications that this flake is monolayered, based on the transmission signal but presents a reduced electron emission compared to other monolayered MXene flakes. This flake presents a constant offset in OD compared to the monolayered violet‐dotted flake. This may be attributed to the existence of a thin organic film below or above the flake that does not contain titanium but other elements absorbing below the Ti L‐edge, such as carbon or nitrogen. The violet‐dotted flake exhibits a higher TEY signal than the red‐dotted flake (Figure 3d), most probably because of better electrical contact with the substrate and the other MXene flakes. The complete data set for a few‐layered MXene at Ti L‐edge is presented in Figure S6 (Supporting Information) and equivalent measurements at O K‐edge are included in Figure S7 (Supporting Information).

Normalization of the XA spectra for at least 20 layers leads to the same XA spectrum, allowing accurate layer estimation for the few‐layered MXene. On the contrary, the normalized spectra for multilayered MXene do not lead to the same spectrum, because of the saturation due to increased thickness. The saturation effect does not hinder the acquisition of discrete peaks at Ti L‐edge in the thinner areas (Cluster 3 of Figure 2b) of multilayered MXene, but it does not allow an accurate estimation of maximum OD. This is best shown for Cluster 1 in Figure 2c, corresponding to the areas with the highest thickness as shown by the fully saturated spectrum, whose maximum OD is decreased rather than increased with thickness compared to Clusters 2 and 3.

### 3.2 Surface Chemistry of HF‐ and MS‐Etched Ti_3_C_2_T_x_ MXene

**Figure 4:**
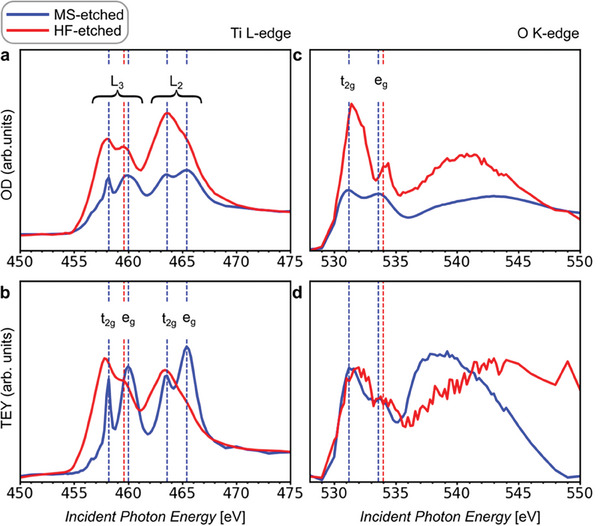
Comparative analysis of normalized transmission and TEY XA spectra for HF‐ and MS‐etched Ti_3_C_2_T_x_ MXene at the Ti L‐edge (a,b) and O K‐edge (c,d). To mitigate the impact of absorption saturation distortion, the spectra displayed for the MS‐etched Ti_3_C_2_T_x_ MXene have been selectively extracted from regions of reduced thickness. Both OD and TEY spectra are normalized.

The multilayered MS‐etched Ti_3_C_2_T_x_ MXene sample (blue spectra in **Figure** **4**a,b) exhibits significant similarities in XA spectra at the Ti L‐edge compared to the delaminated HF‐etched Ti_3_C_2_T_x_ MXene sample (Figure 3 and red spectra in Figure 4a,b). The splitting of the Ti L_2_ and L_3_ components is also apparent for the MS‐etched Ti_3_C_2_T_x_ MXene in both transmission (Figure 4a) and TEY (Figure 4b) modes. On the other hand, the L_2_ *e_g_* peak for the HF‐etched Ti_3_C_2_T_x_ MXene is not pronounced. A noteworthy difference is presented by the L_3_ *e_g_* peak, which is shifted +0.4 eV for the MS‐etched MXene compared to its counterpart in the HF‐etched sample, indicated with a dashed blue line.

Hu et al.^[^ ^36^ ^]^ reported that Ti_3_C_2_T_2_ MXene presents a distorted octahedral symmetry. Ti atoms are in the center of the octahedron, built by terminations and carbon atoms, and the distortion is explained by the different interaction of Ti 3d orbitals with C 2p orbitals and with the orbitals of surface terminations (F, OH, Cl, O). The spectra at the Ti L‐edge (Figure 4a,b) reveal such distortions for both MS‐etched and HF‐etched Ti_3_C_2_T_x_ MXene. The broadening of the *e_g_* peaks in comparison to the *t*_2g_ peaks, observed for both MXenes, can be explained by the more extensive hybridization of Ti atoms with the surface termination groups. The rationale behind this is that the *e*_g_ orbitals are directed toward the ligands, unlike the *t*_2g_ orbitals, which are oriented between the ligands.^[^ ^37^, ^38^ ^]^ The L_2_ edge shows more pronounced broad features, a consequence of the reduced lifetime of the 2p_1/2_ core holes and the near Coster‐Krönig decay process.^[^ ^39^ ^]^ This explains why the *e*_g_ feature is discernible at the Ti L_3_‐edge, and not at the Ti L_2_‐edge for HF‐etched MXene.

HF‐etched MXene has more F‐ and OH‐terminations and fewer O‐terminations than the MS‐etched MXene due to the different etching routes.^[^ ^40^, ^41^ ^]^ The fluorine and hydroxyl terminations of HF‐etched MXene occupy different sites than the bridging oxygen groups, affecting differently the symmetry of MXene.^[^ ^36^ ^]^ For the MS‐etched Ti_3_C_2_T_x_ MXene, the increased *e*_g_ peak at the Ti L‐edge correlates with a higher Ti oxidation state and increased content of oxygen surface terminations. This trend is observed both in the bulk and at the surface of the MXene flake (blue spectra in Figure 4a,b). Splitting of the *e_g_* peak is reported for anatase and rutile titania due to the Jahn‐Taller effect^[^ ^42^ ^]^ which is slightly distorting the octahedral symmetry for MS‐etched MXene.

For the HF‐etched MXene, the reduced *e_g_* peak at the Ti L‐edge correlates with a reduced Ti oxidation state and significant distortion from the octahedral symmetry. Thus, we attribute the change in the Ti oxidation state to the existence of different terminations in HF‐ and MS‐etched MXene. The observed increased Ti oxidation state for MS‐etched MXene and the significantly distorted octahedral symmetry of HF‐etched MXene align with the reported Bader charge analysis for Ti_3_C_2_O_2_ and Ti_3_C_2_F_2_ MXene,^[^ ^36^ ^]^ respectively.

The XAS at the O K‐edge (Figure 4c,d) corresponds to transitions from O 1s core levels to O 2p levels, hybridized with Ti 3d states.^[^ ^37^ ^]^ The peaks at the O K‐edge, labeled as *t*_2g_ and *e*_g_, are the transitions from O 1s to the hybridized orbital of O 2p with Ti 3d *t*_2g_ and *e*_g_, respectively. They are located at 531.2 (531.4) and 533.6 (534.2) eV with an *e*_g_ to *t*_2g_ peak intensity ratio of 0.98 (0.92) for the MS‐etched (HF‐etched) Ti_3_C_2_T_x_ MXene in transmission mode (Figure 4b). The *e*_g_ peak is detected at 0.6 eV higher energy for the HF‐etched MXene.

Figure 4d presents the TEY spectrum at the O K‐edge for both MS‐ and HF‐etched MXenes, with the peaks at the same energy position for the MS‐etched MXene in both detection modes. The quantitative interpretation of the TEY measurements, especially at the O K‐edge, is complex due to the charging phenomenon and carbon deposition during the measurement process (Figures S7 and S9, Supporting Information).

Transmission and TEY XA spectra at Ti L‐ and O K‐edge evidence the differences in distortion of the octahedral symmetry for Ti_3_C_2_T_x_ MXene, caused by different surface termination groups. The higher deviation from the octahedral symmetry is encountered in HF‐etched MXene, most likely due to the high content of F‐terminations having another symmetry. We attribute the increased Ti oxidation state for MS‐etched to bonding between surface Ti atoms and bridging O atoms, rather than electronegative F and OH atoms.

### 3.3 Surface and Bulk Responses of Postmortem Cycled MXene Anode in Li‐Ion Batteries

**Figure 5:**
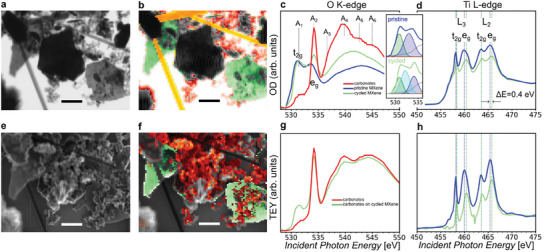
SXM data for cycled MS‐etched Ti_3_C_2_T_x_ MXene. a) Transmission image at the O K‐edge, along with (b) the clustered image and (c) corresponding transmission XA spectra. e) TEY image at the O K‐edge, along with (f) the clustered image and (g) corresponding TEY spectra. Areas of MXene (green), separator (yellow), and electrolyte residues (red) are shown. c) Comparative analysis at the Ti L‐edge in (d) both transmission and (h) TEY modes for pristine and cycled MS‐etched Ti_3_C_2_T_x_ MXene. The spectra at O K‐ and Ti L‐edge of pristine MS‐etched Ti_3_C_2_T_x_ MXene (blue) are added for comparison. An inset with fitted spectra of the pre‐edge of pristine and cycled MXene is included. Scalebar: 2 µm.

The postmortem SXM analysis of MS‐etched Ti_3_C_2_T_x_ MXene electrodes after cycling in a classical Li‐ion battery (2 cycles from 3 to 0 V) was attempted to identify the change of MXene chemical bonding after cycling. The SXM images averaged over the O K‐edge are shown in **Figure** **5**a,b,e,f. Battery components shown in Figure 5b, such as cycled MXene electrodes (green), separator (yellow), and carbonate species (red), are imaged with their corresponding transmission XA spectra in Figure 5c. Simultaneously, the TEY image for the same area is also averaged over the energy range of the O K‐edge (Figure 5e). Red‐colored areas cover almost fully the MXene (Figure 5f), leading to weak detection of MXene in TEY mode (Figure 5g).

The XA spectra at the O K‐edge can be described by 6 components, labeled A_1‐6_ which are visible in both detection modes (Figure 5c,g), allowing the chemical identification of the various components of the dismounted cell. A_1_ peak, located at 531.4 eV, is attributed to the *t*_2g_ peak of Ti_3_C_2_T_x_ MXene. In TEY XA spectra (Figure 5g), the A_1_ peak is discernible in areas where the surface of MXene is not fully covered with other battery components (Figure S14, Supporting Information). A_2_ peak, located at 534.2 eV, is close to the *e*_g_ peak of Ti_3_C_2_T_x_ MXene, but it is shifted by +0.6 eV compared to pristine Ti_3_C_2_T_x_ MXene.

The peaks A_2_ (534.2 eV), A_3_ (536.6 eV), A_4_ (539.8 eV), and A_6_ (544 eV) are related to the transition to different CO bonds coming from the solid electrolyte interface (SEI), electrolyte residues and other carbonate species.^[^ ^43^, ^44^, ^45^ ^]^ Sharp peaks at the O K‐edge without any trace of MXene are presented in Figure S13 (Supporting Information). Wang et al.^[^ ^46^ ^]^ reports that Li_2_CO_3_ is the main component of the SEI layer, as a result of interaction between lithiated titania with the electrolyte. The red transmission XA spectrum (Figure 5c), and especially the overall TEY XA spectrum (Figure 5g) at O K‐edge are very similar to the TEY‐XA spectra reported for intercalated V_2_CT_x_ MXene.^[^ ^47^ ^]^ We assign the A_2_ peak to CO_3_^2−^ species, related to the interaction of cycled Ti_3_C_2_T_x_ MXene and carbon‐based electrolyte.

The changes in the components of the carbonate species are further analyzed by peak fitting of all spectra at the O K‐edge (Figure S14, Supporting Information). Indeed, we notice that the red areas are mainly described by the A_2_, A_3_, A_4,_ and A_6_ components related to carbonate species, which dominate the signal in TEY mode (Figure 5g).

The understanding of the possible interaction of MXene's bulk flake with intercalated species during cycling is enabled by comparing the transmission spectrum of cycled MXene (green) to the pristine MXene (blue) and carbonate species (red) (Figure 5c). Two main changes in the transmission spectrum XAS at the O K‐edge of cycled and pristine MXene are observed: i) the A_1_ peak in cycled MXene is broader compared to the peak in the pristine MXene, and ii) the increased A_2_ peak in cycled MXene, which is the contribution of the top layers of the sample to the transmission spectrum. An additional peak at 532.2 eV is required for a good fit of the XA spectrum for cycled MXene (Figure S14, Supporting Information). We tentatively assign this peak, highlighted with cyan in the inset of Figure 5, to Li cations interacting during cycling with O‐terminations. Such broadening has also been reported for Li‐ and Na‐ pre‐intercalated Ti_3_C_2_T_x_ MXenes.^[^ ^48^ ^]^ Regardless, the changes between pristine and cycled MXene are not dramatic, indicating that the MXene structure remains stable during cycling.

The transmission spectra at the Ti L‐edge inform about the change in the Ti oxidation state and overall symmetry of cycled MXene's upper and inner layers. The transmission and TEY XA spectra are obtained for pristine (blue) and cycled (green) Ti_3_C_2_T_x_ MXene (Figure 5d,h, respectively). The L_3_(L_2_) *t*_2g_ and *e*_g_ peak for cycled MXene are located at 458.4 eV (463.6 eV) and 460.4 eV (465.8 eV), respectively for both transmission and TEY mode. The sensitivity of the *e_g_* peak to the environment of MXene is remarkable. The *e*_g_‐to*‐t*_2g_ peak intensity ratio is higher in TEY mode than in transmission mode, which can be interpreted by the surface being more oxidized than the bulk (increased Ti^4+^/Ti^3+^ ratio), probably due to exposure of the MXene's upper layers to the electrolyte and/or air.

Differences of XA spectra at Ti L‐edge for pristine (blue) and cycled (green) MXene in both transmission and TEY mode are observed. All peaks are shifted to higher photon energy for cycled compared to pristine MXene. Additionally, the Ti L *e*_g_ peak of cycled MXene is 0.4 eV higher than the one of pristine MXene. Koudriachova et al.^[^ ^49^ ^]^ reports distortion of the octahedral symmetry upon Li^+^ intercalation for titania. Such distortion can lead to increased Ti L *e*_g_ peak toward higher energy, as shown with MXenes with ligands, requiring an increased Ti oxidation state to stabilize the MXene. We assume that this increased Ti oxidation state in cycled MXene is related to the surface Ti atoms with O‐terminations interacting with lithium cations.

In summary, the electrochemical cycling of MXene electrode in Li‐ion battery does not lead to a dramatic change of the MXene structure, but rather further distortion of the octahedral symmetry for cycled MS‐etched MXene. Electronic interaction between Li^+^ cation and oxygen orbitals of the MXenes interlayer chemistry is evidenced, as exhibited by the difference in transmission spectra at Ti L‐ and O K‐edge between pristine and cycled MXene. Therefore, we claim that the changes in the bulk of the cycled MXene are related to Li^+^ bonding with O‐terminations. TEY spectrum at O K‐edge locates the carbonate species on top of the MXene. All the regions of the dismounted cell and the corresponding data analysis are presented in Figures S11–S14 (Supporting Information).

## 4 Conclusion

Ti_3_C_2_T_x_ MXenes flakes, ranging from mono‐ to multilayers, were imaged using multimodal SXM with a spatial resolution of ˂50 nm. Simultaneous hyperspectral X‐ray imaging in transmission and TEY modes provides insights into the local chemistry of the MXene surface and bulk regions on individual flakes. SXM allows the monitoring of MXene flake thickness and electron emission properties at the sub‐flake level. Local XAS measurements have a high chemical sensitivity to the Ti and O bonding configurations, hence to the surface chemistry of MXene. The Ti chemical bonding differs dramatically between HF‐etched and MS‐etched Ti_3_C_2_T_x_ MXenes explained by the larger content of O‐termination in MS‐Ti_3_C_2_T*_x_*. Postmortem analysis of an MXene‐based Li‐ion battery allows identification of both pristine and electrochemically cycled Ti_3_C_2_T_x_ MXene. Upon comparing pristine and cycled MXene, we discern signs of a chemical interaction between Li^+^ and the MXene flakes. Furthermore, surface‐sensitive TEY measurements hint at surface reactions between the MXene and the electrolyte during the electrochemical cycling process. This distinction underscores the complex interplay of bulk intercalation and surface phenomena in these materials. Multimodal SXM can be applied to other laminated materials, such as graphene, layered transition metal dichalcogenides (TMDs), thin film solar cells, and multilayered polymer films, thereby opening new insights into intercalation and surface redox processes.

## 5 Experimental Section

### SXM Imaging

The measurements were performed at the ultra‐high vacuum scanning X‐ray microscopy (UHV‐SXM) “MAXYMUS” microscope endstation at the UE46‐PGM2 undulator beamline at HZB/BESSY II. Surface‐sensitive total electron yield (TEY) measurements were conducted simultaneously with standard transmission measurements by amplifying the sample current using a commercial amplifier from FEMTO Messtechnik GmbH, and then converting it to corresponding frequency values. To boost the total sample current, the OSA – a device was set that prevents zero‐order light from hitting the sample – to a positive bias voltage of ≈+ 30 V. This approach reduced the number of unwanted electrons released from the Fresnel ZP due to incoming X‐ray excitation. The transmission X‐ray flux was recorded using an avalanche photodiode.

### Sample Preparation

SiN membranes (Silson) with high crystallinity for both transmission and TEY experiments were used for this study. The membrane size was 1 mm × 1 mm with a thickness of 100 nm, and the Si frame size was 5 mm × 5 mm with a thickness of 200 µm.

### MXene Synthesis

Two types of MXenes were used for this study: few‐layered HF‐etched Ti_3_C_2_T_x_ MXenes and multilayered molten salt (MS)‐etched Ti_3_C_2_T_x_ MXenes. The synthesis of multilayered Ti_3_C_2_T_x_ MXene powders, used for HF‐etched Ti_3_C_2_T_x_ MXene, followed the procedure proposed by Mathis et al.^[^ ^40^ ^]^ Briefly, one gram of MAX powder was slowly added to a mixture of 6 mL H_2_O, 12 mL (37 wt.%) HCl, and 2 mL (49 wt.%) HF in a vented polytetrafluoroethylene bottle. The solution was stirred at 300 rpm for 24 h at 35 °C. The resulting MXene powder was then centrifuged at 2500 RCF for 5 min in a 175 mL tube, and the clear supernatant was decanted. This centrifugation and decantation process was repeated until the supernatant reached a pH of ≈6. Multilayer MXene in LiCl solution was stirred and settled, and the supernatant was collected. After refilling with water, MXene redispersion was done multiple times until a transparent supernatant indicated a low MXene concentration. LiCl concentration decrease led to spontaneous MXene delamination, forming a stable, darker colloidal solution, as reported in the reference.^[^ ^50^ ^]^ The XRD pattern of the MAX phase, etched and delaminated HF‐Ti_3_C_2_T_x_ MXenes is shown in Figure S15 (Supporting Information).

Molten Salt‐Shielded Synthesis (MS^3^) of MXenes in the air involves the preparation of MXenes via the molten salt‐shielded synthesis method as previously reported.^[^ ^41^ ^]^ Specifically, 1 g of Ti_3_AlC_2_ MAX phase powder was combined with a eutectic salt (NaCl:KCl in a 1:1 molar ratio) in a weight ratio between 1:6. This blend was ground for 10 min using a mortar and pestle. The ground powder was then subjected to uniaxial pressure in a 20 mm diameter steel die under a load of 50 kg cm^−2^ to form a pellet. This formed pellet was then placed in a cylindrical alumina crucible. Next, a mixture of salts (8.7 g of NaCl, 11.2 g of KCl, and 4 g of CuCl_2_) was added to a 30 mL crucible to cover the pellet following 10 min of milling. This crucible was then sealed with an alumina lid and positioned in a muffle furnace. The furnace was heated to 700 °C at a rate of 10 °C per minute and maintained at this temperature for 40 min. After the furnace cooled to room temperature, the Ti_3_C_2_T_x_/Cu mixture was repeatedly rinsed with deionized water to rid it of salts. The Cu from the resultant Ti_3_C_2_T_x_ /Cu mixture was then eliminated by washing it with 100 mL of 0.5 MAPS solution for 1 h at room temperature. This solution was then washed more than five times with deionized water and filtered. Finally, the Ti_3_C_2_T_x_ MXene powder was left to dry under vacuum at room temperature for 12 h (see XRD pattern in Figure S15, Supporting Information). The chlorine termination cannot be probed directly with STXM in the soft X‐ray range. However, oxygen terminal groups are more stable than chlorine ones,^[^ ^51^ ^]^ which explains the high oxygen content observed in MS‐Ti_3_C_2_T_x_.

### Electrochemical Cycling

Two‐electrodes Swagelok cell was used for the electrochemical measurement, with the working electrode composed of MS‐Ti_3_C_2_T_x_ MXene powder mixed with carbon black with a weight ratio of 9:1. Metallic lithium foil is used as counter electrode, reference electrode and LP30 (1 m LiPF_6_ in 1:1 vol/vol ethylene carbonate/dimethyl carbonate) as the electrolyte, and Whatman glassy fiber GF/A was used as separator. Cyclic voltammetry tests (Figure S10, Supporting Information) were conducted in the potential range of 0.2–3 V versus Li^+^/Li, with a scan rate of 0.5 mV s^−1^. Upon reduction during the first cycle, the formation of a solid electrolyte interphase (SEI) layer is expected.

Post‐cycling, the MXene powder was recovered from the cell and rinsed with dimethyl carbonate (DC) to remove excess electrolytes. Following sample loading into the SXM hatch, the DC was evaporated due to the high vacuum conditions.

## Conflict of Interest

The authors declare no conflict of interest.

## Author Contributions

T.P. conceptualized and supervised the project. P.B. performed all the SXM experiments with help from F.A. and M.W. F.A. performed all the data analysis under the supervision of T.P. H.S. synthesized the multilayered Ti_3_C_2_T_x_ MXene and performed electrochemical cycling. P.B. synthesized the few‐layered Ti_3_C_2_T_x_ MXene, with the MAX precursor that J.G.J provided. F.A. and T.P. wrote the manuscript with contributions from the authors.

## Supporting information

Supporting Information

## Data Availability

The data that support the findings of this study are available in the supplementary material of this article.

## References

[1] Y. Ding , M. Zeng , Q. Zheng , J. Zhang , D. Xu , W. Chen , C. Wang , S. Chen , Y. Xie , Y. Ding , S. Zheng , J. Zhao , P. Gao , L. Fu , Nat. Commun. 5886, 12, 2021. 34620848 10.1038/s41467-021-26139-5 PMC8497624

[2] K. D. Rasamani , F. Alimohammadi , Y. Sun , Mater. Today 2017, 20, 83.

[3] C. Yin , Z. Wei , M. Zhang , B. Qiu , Y. Zhou , Y. Xiao , D. Zhou , L. Yun , C. Li , Q. Gu , W. Wen , X. Li , X. Wen , Z. Shi , L. He , Y. S. Meng , Z. Liu , Mater. Today 2021, 51, 15.

[4] M. Rajapakse , B. Karki , U. O. Abu , S. Pishgar , M. R. K. Musa , S. M. S. Riyadh , M. Yu , G. Sumanasekera , J. B. Jasinski , npj 2D Materials and Applications 2021, 5, 30.

[5] C. Yin , C. Pan , X. Liao , Y. Pan , L. Yuan , ACS Appl. Mater. Interfaces 2021, 13, 39347. 34383482 10.1021/acsami.1c09722

[6] W. B. Johnson , W. L. Worrell , Synth. Met. 1982, 4, 225.

[7] Y. Gu , Y. Katsura , T. Yoshino , H. Takagi , K. Taniguchi , Sci. Rep. 2015, 5, 12486. 26228263 10.1038/srep12486 PMC4521182

[8] B. Anasori , M. R. Lukatskaya , Y. Gogotsi , Nat. Rev. Mater. 2017, 2, 16098.

[9] Z. Liu , H. N. Alshareef , Adv. Electron. Mater. 2021, 7, 2100295.

[10] M. A. K. Purbayanto , M. Chandel , M. Birowska , A. Rosenkranz , A. M. Jastrzębska , Adv. Mater. 2023, 35, 2301850. 10.1002/adma.202301850 37715336

[11] M. Han , D. Zhang , C. E. Shuck , B. McBride , T. Zhang , R. (John) Wang , K. Shevchuk , Y. Gogotsi , Nat. Nanotechnol. 2023, 18, 373. 36646826 10.1038/s41565-022-01308-9

[12] K. Rasool , R. P. Pandey , P. A. Rasheed , S. Buczek , Y. Gogotsi , K. A. Mahmoud , Mater. Today 2019, 30, 80.

[13] H.‐J. Koh , S. J. Kim , K. Maleski , S.‐Y. Cho , Y.‐J. Kim , C. W. Ahn , Y. Gogotsi , H.‐T. Jung , ACS Sens. 2019, 4, 1365. 31062965 10.1021/acssensors.9b00310

[14] Y. Dong , H. Shi , Z.‐S. Wu , Adv. Funct. Mater. 2020, 30, 2000706.

[15] T. Koriukina , A. Kotronia , J. Halim , M. Hahlin , J. Rosen , K. Edström , L. Nyholm , ACS Omega 2022, 7, 41696. 36406498 10.1021/acsomega.2c05785 PMC9670687

[16] M. M. Baig , I. H. Gul , S. M. Baig , F. Shahzad , J. Electroanal. Chem. 2022, 904, 115920.

[17] C. Zeng , J. Liang , C. Cui , T. Zhai , H. Li , Adv. Mater. 2022, 34, 2200777. 10.1002/adma.202200777 35363408

[18] M. Naguib , M. Kurtoglu , V. Presser , J. Lu , J. Niu , M. Heon , L. Hultman , Y. Gogotsi , M. W. Barsoum , Adv. Mater. 2011, 23, 4248. 21861270 10.1002/adma.201102306

[19] Y. Li , H. Shao , Z. Lin , J. Lu , L. Liu , B. Duployer , P. O. Å. Persson , P. Eklund , L. Hultman , M. Li , K. Chen , X. H. Zha , S. Du , P. Rozier , Z. Chai , E. Raymundo‐Piñero , P. L. Taberna , P. Simon , Q. Huang , Nat. Mater. 2020, 19, 894. 32284597 10.1038/s41563-020-0657-0

[20] J. Björk , J. Rosen , Chem. Mater. 2021, 33, 9108.

[21] H. Tan , J. Verbeeck , A. Abakumov , G. Van Tendeloo , Ultramicroscopy 2012, 116, 24.

[22] A. Sarycheva , M. Shanmugasundaram , A. Krayev , Y. Gogotsi , ACS Nano 2022, 16, 6858. 35404582 10.1021/acsnano.2c01868

[23] Y. Wu , D. Li , C.‐L. Wu , H. Y. Hwang , Y. Cui , Nat. Rev. Mater. 2023, 8, 41.

[24] S. I. Bokarev , O. Kühn , WIREs Computational Molecular Science 1433, 10, e2020.

[25] B. Wu , B. Wang , T. Petit , Energy Storage Mater. 2021, 40, 72.

[26] K. Wojtaszek , W. Błachucki , K. Tyrała , M. Nowakowski , M. Zając , J. Stępień , P. Jagodziński , D. Banaś , W. Stańczyk , J. Czapla‐Masztafiak , W. M. Kwiatek , J. Szlachetko , A. Wach , The Journal of Physical Chemistry A 2021, 125, 50. 33395294 10.1021/acs.jpca.0c07955

[27] A. Al‐Temimy , F. Kronast , M.‐A. Mawass , K. A. Mazzio , K. Prenger , M. Naguib , T. Petit , S. Raoux , Appl. Surf. Sci. 2020, 530, 147157.

[28] A. Al‐Temimy , B. Anasori , K. A. Mazzio , F. Kronast , M. Seredych , N. Kurra , M.‐A. Mawass , S. Raoux , Y. Gogotsi , T. Petit , J. Phys. Chem. C 2020, 124, 5079.

[29] M. Mirolo , D. Leanza , L. Höltschi , C. Jordy , V. Pelé , P. Novák , M. El Kazzi , C. A. F. Vaz , Anal. Chem. 2020, 92, 3023. 31961659 10.1021/acs.analchem.9b04124

[30] S. Spence , W.‐K. Lee , F. Lin , X. Xiao , Nanotechnology 2021, 32, 442003. 10.1088/1361-6528/ac17ff 34315146

[31] B. Kaulich , P. Thibault , A. Gianoncelli , M. Kiskinova , J. Phys.: Condens. Matter 2011, 23, 083002. 21411893 10.1088/0953-8984/23/8/083002

[32] D. Nolle , M. Weigand , G. Schütz , E. Goering , Microsc. Microanal. 2011, 17, 834. 21864447 10.1017/S1431927611000560

[33] R. Nakajima , J. Stöhr , Y. U. Idzerda , Phys. Rev. B 1999, 59, 6421.

[34] J. G. Chen , J. Eng , S. P. Kelty , Catal. Today 1998, 43, 147.

[35] E. Stoyanov , F. Langenhorst , G. Steinle‐Neumann , Am. Mineral. 2007, 92, 577.

[36] T. Hu , Z. Li , M. Hu , J. Wang , Q. Hu , Q. Li , X. Wang , J. Phys. Chem. C 2017, 121, 19254.

[37] A. Gloter , C. Ewels , P. Umek , D. Arcon , C. Colliex , Phys. Rev. B 2009, 80, 035413.

[38] K. Płacheta , A. Kot , J. Banas‐Gac , M. Zając , M. Sikora , M. Radecka , K. Zakrzewska , Appl. Surf. Sci. 2023, 608, 155046.

[39] F. M. F. de Groot , H. Elnaggar , F. Frati , J. Electron Spectrosc. Relat. Phenom. 2021, 249, 147061.

[40] T. S. Mathis , K. Maleski , A. Goad , A. Sarycheva , M. Anayee , A. C. Foucher , K. Hantanasirisakul , C. E. Shuck , E. A. Stach , Y. Gogotsi , ACS Nano 2021, 15, 6420. 33848136 10.1021/acsnano.0c08357

[41] J. Chen , Q. Jin , Y. Li , H. Shao , P. Liu , Y. Liu , P.‐L. Taberna , Q. Huang , Z. Lin , P. Simon , Energy & Environmental Materials 2023, 6, e12328.

[42] R. Brydson , B. G. Williams , W. Engel , H. Sauer , E. Zeitler , J. M. Thomas , Solid State Commun. 1987, 64, 609.

[43] M. Schellenberger , R. Golnak , W. G. Quevedo Garzon , S. Risse , R. Seidel , Materials Today Advances 2022, 14, 100215.

[44] Swallow, J. E. N. , M. W. Fraser , N.‐J. H. Kneusels , Nat. Commun. 2022, 13, 6070. 36241622 10.1038/s41467-022-33691-1 PMC9568580

[45] Qiao, R. , Chuang, Y. D. , Yan, S. , Yang, W. , PLoS One 2012, 7, e49182. 23145116 10.1371/journal.pone.0049182 PMC3492314

[46] D. Wang , L. Liu , X. Sun , T.‐K. Sham , J. Mater. Chem. A 2015, 3, 412.

[47] S. M. Bak , R. Qiao , W. Yang , S. Lee , X. Yu , B. Anasori , H. Lee , Y. Gogotsi , X.‐Q. Yang , Adv. Energy Mater. 2017, 7, 1700959.

[48] A. Al‐Temimy , K. Prenger , R. Golnak , M. Lounasvuori , M. Naguib , T. Petit , ACS Appl. Mater. Interfaces 2020, 12, 15087. 32134245 10.1021/acsami.9b22122

[49] M. V. Koudriachova , S. W. de Leeuw , N. M. Harrison , Phys. Rev. B 2004, 69, 054106.

[50] M. Shekhirev , J. Busa , C. E. Shuck , A. Torres , S. Bagheri , A. Sinitskii , Y. Gogotsi , ACS Nano 2022, 16, 13695. 35877963 10.1021/acsnano.2c04506

[51] P. Liu , P. Xiao , M. Lu , H. Wang , N. Jin , Z. Lin , Chin. Chem. Lett. 2023, 34, 107426.

