# Supplementary material for: Nanoscale Surface and Bulk Electronic Properties of Ti3C2Tx MXene Unraveled by Multimodal X‐Ray Spectromicroscopy
Source: Small Methods. 2024 Jun 14;8(12):2400190. doi: 10.1002/smtd.202400190 (PMC11671855; doi:10.1002/smtd.202400190)
Supplement: Supplementary file 1 — Supporting Information [file SMTD-8-2400190-s001.docx]

Supporting Information

Nanoscale surface and bulk electronic properties of Ti_3_C_2_T_x_ MXene unraveled by multimodal X-ray spectromicroscopy

Faidra Amargianou^1,2^, Peer Bärmann^1^, Hui Shao^3^, Pierre-Louis Taberna^3^, Patrice Simon^3^, Jesus Gonzalez-Julian^4^, Markus Weigand^1^, and Tristan Petit^1,^*

^1^ Helmholtz-Zentrum Berlin für Materialien und Energie GmbH, Albert-Einstein-Straße 15, 12489 Berlin, Germany

^2^ Faculty of Mathematics and Natural Sciences, TU-Berlin, Hardenbergstr. 36, 10623 Berlin, Germany

^3^ Université Paul Sabatier, CIRIMAT UMR CNRS 5085, 118 route de Narbonne, 31062 Toulouse, France

^4^ Institute of Mineral Engineering (GHI), Chair of Ceramics, RWTH Aachen, 52074 Aachen, Germany

*E-mail: [tristan.petit@helmholtz-berlin.de](mailto:tristan.petit@helmholtz-berlin.de)

**Table of Content**

[1. Information depth of transmission and total electron yield detections 2](#_Toc168902825)

[2. Cluster analysis 3](#_Toc168902826)

[3. Estimation of the optical density for a monolayer flake 6](#_Toc168902827)

[4. Few-layered HF-etched Ti_3_C_2_T_x_ MXene 7](#_Toc168902828)

[5. Physical effects in transmission and TEY 9](#_Toc168902829)

[6. Electrochemically cycled MS- etched Ti_3_C_2_T_x_ MXene 12](#_Toc168902830)

[7. X-ray Diffraction patterns of MS- and HF-Ti_3_C_2_T*_x_* MXenes 17](#_Toc168902831)

[8. References 17](#_Toc168902832)

## 1. Information depth of transmission and total electron yield detections

X-ray absorption measurements, conducted in transmission, are directly proportional to the number of core holes produced during absorption. Absorption coefficients (µ) are obtained from transmission measurements, described by Beer-Lambert’s law:

$I\left( hv \right)=I_{0}e^{-\mu t}$ (1)

Here, I and I_0_ represent the intensity of the transmitted and incoming photons, respectively. µ is the linear absorption coefficient (a function of photon energy v), and t is the sample thickness. The optical density (OD) is given by $OD=-ln\frac{I}{I_{0}}=\mu t$.

From Equation S1, given the sample thickness, the absorption coefficient can be estimated as $\mu=-\left( 1/t \right)\cdot ln\left( I/I_{0} \right)$ can be estimated given the value of thickness. When saturation effects are observed in the transmission signal (transmission intensity tends to 0), we can assume infinite thickness and set f(t) to 1. If the escape depth is much shorter than the penetration length, then $\lambda_{x}=1/\mu$.

For TEY in the sub-keV soft X-ray range, low-energy secondary electrons primarily contribute to the detected signal^[^[^1^](#ref-Frazer2003TheX-PEEM)^]^. Probing the Ti L-edge with an incident photon energy of 463 eV results in an Auger electron effective range of about 4 nm and a mean probing depth of about 4.7 nm^[^[^1^](#ref-Frazer2003TheX-PEEM)^]^. The information depth is associated with the maximum probing depth, specifically the mean free path of the Auger electron. The detected electrons are primarily the result of secondary electrons scattered inelastically by Auger electrons, leading to a sensitivity of approximately 2 to 4 nm for the Ti L- edge. The electron yield is given by^[^[^2^](#ref-Nakajima1999Electron-yieldNi)^]^:

$Y_{e}\propto Io\cdot\frac{\lambda_{e}}{cos\theta}\cdot f\left( t \right)\cdot\mu\cdot\frac{1}{1+\frac{\lambda_{e}}{\lambda_{x}cos\theta}}$ (2)

Here, *λ_e_* is the electron escape depth, and *λ_x_cosθ* is the X-ray penetration depth, with θ as the angle between the incoming X-rays and the surface normal.

An exponential decay of signal can be expected with increasing thickness of the sample $f\left( t \right)=1-e^{-t\left( \frac{1}{\lambda_{e}}+\frac{1}{\lambda_{x}cos\theta} \right)}$. If the escape depth is much shorter than the penetration depth, as typically the case for single-layered MXenes, the electron yield is also proportional to the absorption coefficient as derived from Equation S2:

$Y_{e}\propto Io\cdot\frac{\lambda_{e}}{cos\theta}\cdot\mu\cdot\left( 1-e^{-\frac{t}{\lambda_{e}}} \right)$ (3)

For TEY measurements, potential effects include saturation (when the escape depth is significantly longer than the penetration depth), edge enhancement (increased θ leading to heightened electron yield), and charging (due to insulated samples or substrates).

## 2. Cluster analysis

Two main techniques were used to determine the major components of the images: Principal Component Analysis (PCA) and Independent Component Analysis (ICA). PCA reduces the dimensionality of data by identifying and retaining the most significant orthogonal directions of variance. On the other hand, ICA distinguishes the dataset into additive subcomponents that are maximally independent, assuming non-Gaussian components. ICA is predominantly used to separate superimposed signals.

Two clustering algorithms, k-means and Gaussian mixture models, were also employed. The k-means algorithm aims to divide samples into *n* groups of equal variance by minimizing a criterion based on Euclidean distances between datapoints or pixels. The Gaussian mixture model fits a mixture of Gaussian models through the expectation-maximization algorithm. When PCA is paired with k-means, the optimal number of clusters can be determined using the elbow method.

For this study, a specific set of images (2*82) of 100 nm by 100 nm per pixel were acquired in both modes at the Ti L-edge across a specified energy range (Ti L-edge: 450-475 eV). Specifically, **Figure** **S1 a** and **b** are images, averaged over the above specified energy range in transmission and TEY mode, respectively. For the estimation of incident intensity I_o_, we select areas without sample. **Figure** **S1 c** is a binary image with black and white regions with and without sample, as created by a threshold value for the I_o_ (**Figure** **S1 d**). The value of I_o_ is estimated for each energy at Ti L-edge by averaging over all the pixels belonging to the white area. The average I_o_ values are plotted versus the photon energy (**Figure** **S1 e**). Then, from the relation OD=-ln(I/I_o_) we extract the OD images at Ti L-edge.


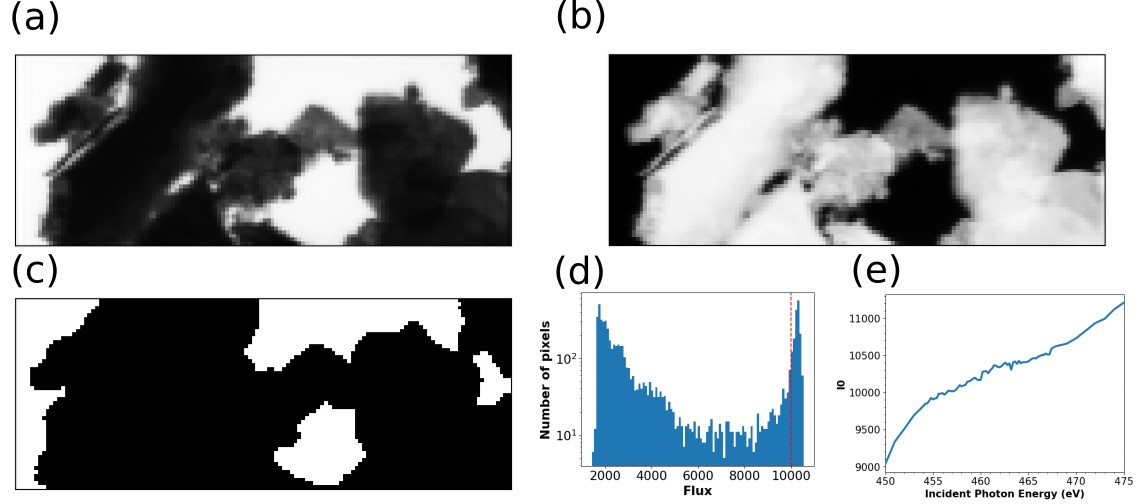


**Figure S1.** Transmission and TEY image, averaged over the energy range from 450 to 475 eV, for pristine multilayered Ti_3_C_2_T_x_ MXene. (c) Binary image, in which the white area is pixels with average flux higher than 10 kcounts. (d) Histogram of the number of pixels versus their average flux. (e) Estimation of the average flux of the pixels with flux higher than 10 kcounts.

We reduce the number of OD images to a few principal components. **Figure S2** presents the first four principal component (PC) images and the corresponding spectra. The first eigenvalue has negative value which is unphysical and therefore does not carry insightful information. Negative eigenvalues in PCA are aligned with the necessity for orthogonal components.


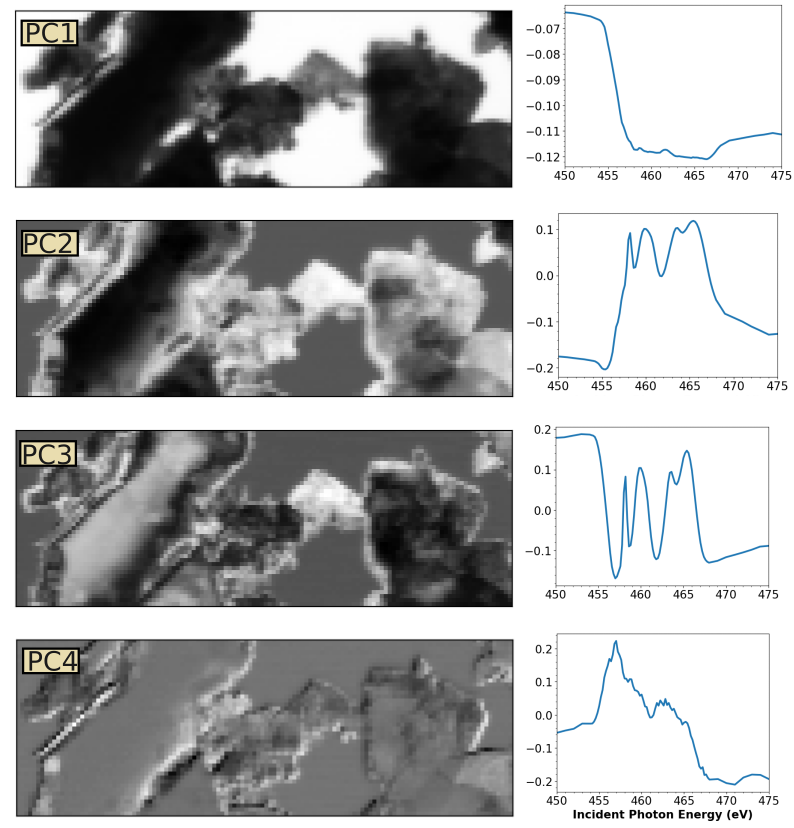


**Figure S2.** First four principal components for pristine multilayered Ti_3_C_2_T_x_ MXene.

We plot the explained variance ratio and ‘Within-Cluster Sum of Square’ against the different values of PCs and clusters to estimate the number of the important ones. The elbow method, despite its occasional ambiguity in determining the exact number of components or clusters, has been utilized to ascertain significant components or clusters. In this context, it has been applied to retain components with a variance higher than 99.9% of the total variance and to identify only two important clusters, corresponding to the MXene sample and the substrate (Figure S3). Nevertheless, the number of clusters was increased to pinpoint the thinner MXene areas and thereby mitigate absorption saturation distortion.

The image and data analysis were performed using customized scripts in Python with libraries such as scikit-learn (<https://scikit-learn.org/stable/>).


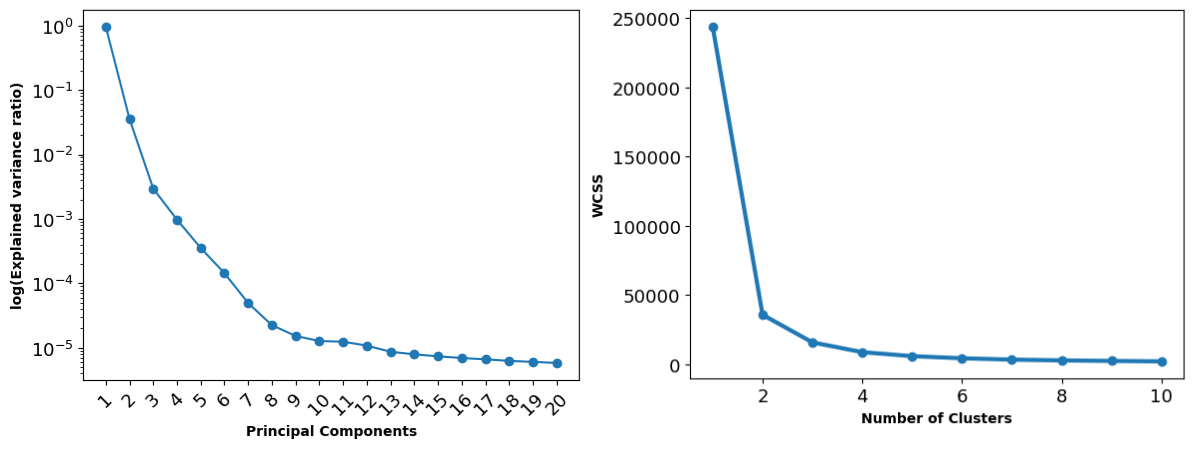


**Figure S3.** Elbow method for the estimation of the number of components and clusters.

## 3. Estimation of the optical density for a monolayer flake


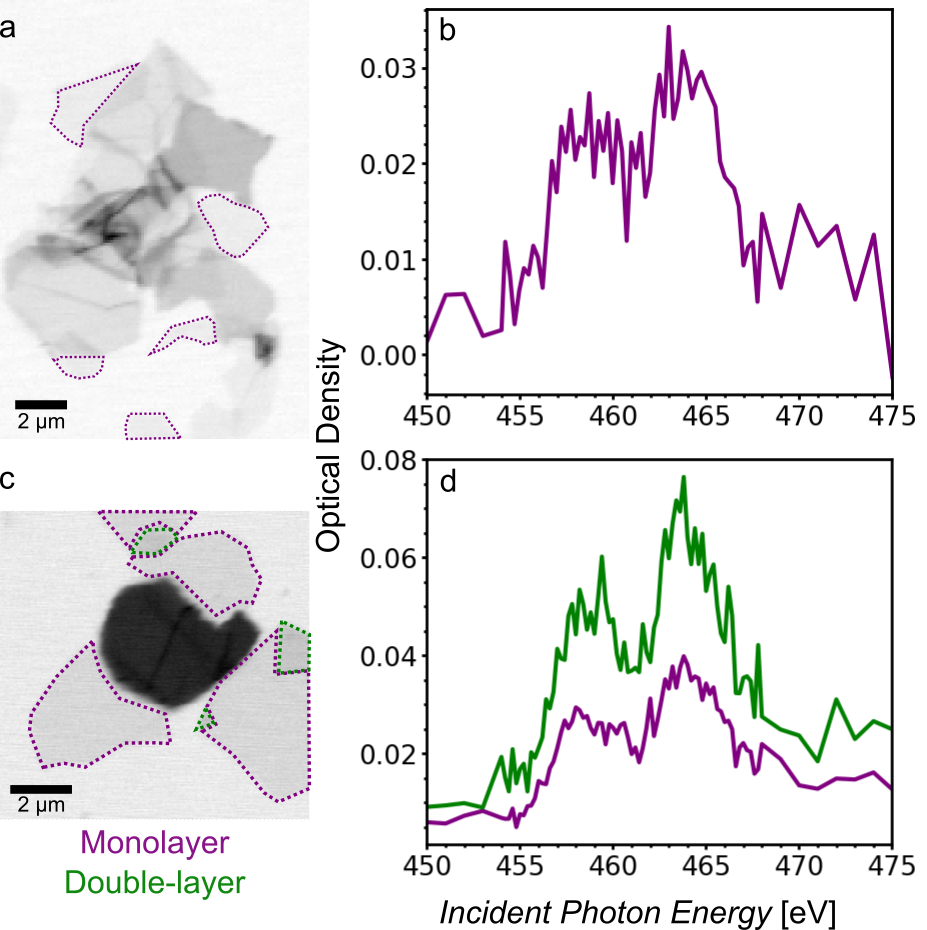


**Figure S4.** **a-c** Transmission images, averaged over the energy range 450-475 eV (Ti L-edge), highlighting regions corresponding to monolayer and double-layer MXene flakes (dotted violet and green, respectively), and **b-d** corresponding spectra of monolayer and double-layer flakes.

Additionally to the SXM images of few-layered MXene flakes (**Figure 3**) with a maximum OD_mono_ of 0.045, we present two examples of few-layered MXene with monolayer flakes. The monolayer flakes, imaged in **Figure S4 a** and **c**, correspond to a violet-coded OD spectra **Figure S4 b-d** with a maximum OD_mono_ of 0.036618 and 0.034838, respectively. In **Figure S4 c**, either the folding of the monolayer flake or the overlapping of monolayer flakes can create regions of double-layered flakes with OD_double_ of 0.067403. There is an error in the estimation of OD_mono_ due to the SXM noise (0.005), estimated by averaging the OD over energies at the pre-edge (450-454.6 eV). Considering all these three examples, we estimate the maximum value of OD_mono_= 0.039 $\pm$ 0.005.

## 4. Few-layered HF-etched Ti_3_C_2_T_x_ MXene

The complete data set of few-layered MXene at Ti L-edge is presented in **Figure S5**. The methods used for defining the clustered areas and spectra in transmission mode are PCA and GMM, while the equivalent in TEY mode is ICA and GMM. Moreover, TEY imaging is very sensitive to the surface morphology of the sample due to the edge enhancement phenomenon. This is presented as high TEY signal in the areas, that the MXene flakes crumple against each other, creating creases and folds, as indicated by orange arrows in **Figure S5**.


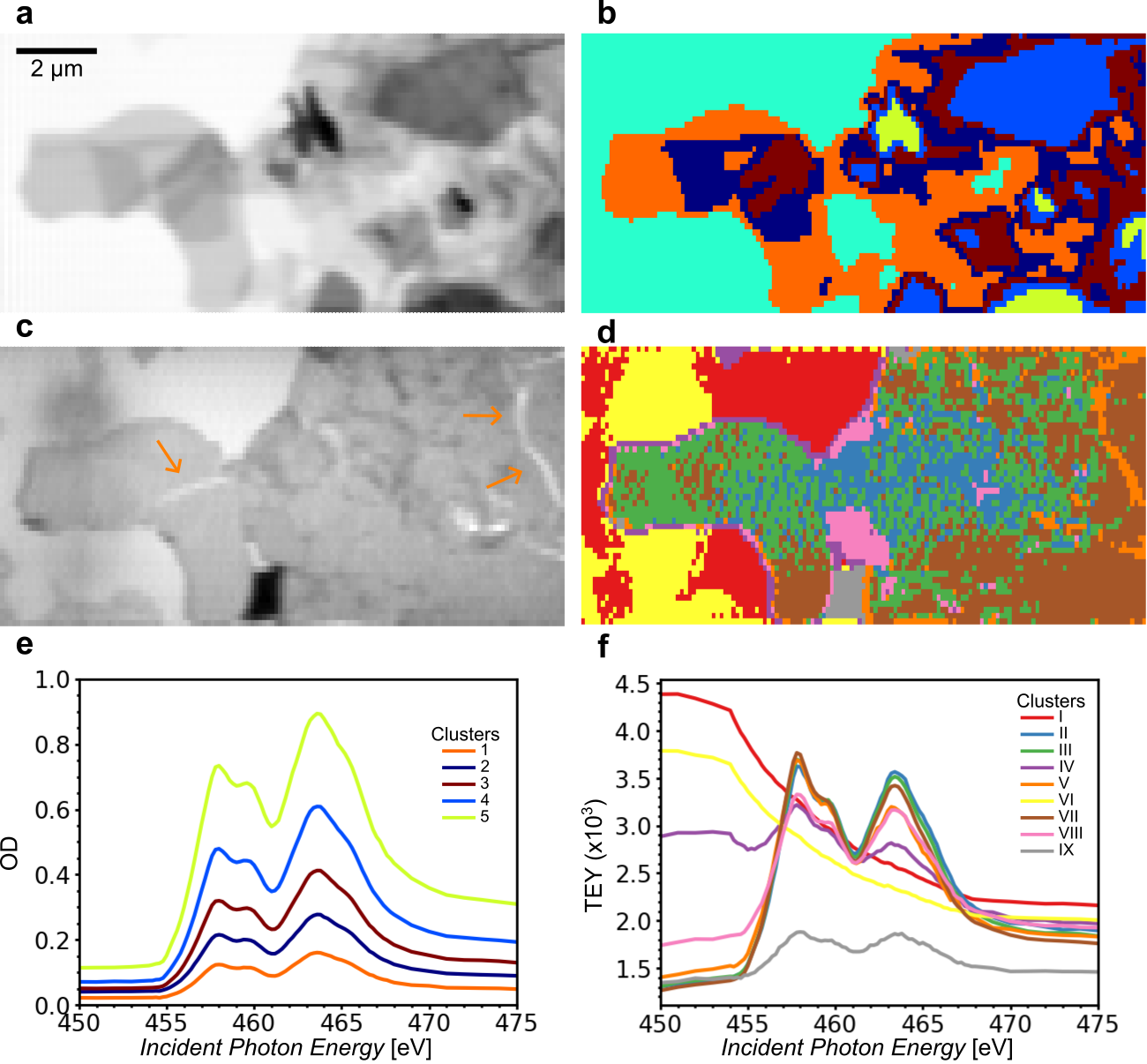


**Figure S5.** High resolution image **a** in transmission and **c** in TEY mode, averaged at Ti L-edge, with corresponding clustered areas (**b-d**) and spectra (**e-f**). PCs, excluding 1st one, of OD dataset are clustered with k-means. ICs, excluding 1st one, of TEY dataset are clustered with GMM.

**Figure S6** presents the transmission image averaged at O K-edge over the energy range of 528-550 eV. The two clustered areas, colored with orange and gray, for transmission measurements identify the thinner and thicker parts of this few-layered Ti_3_C_2_T_x_ MXene sample. These two areas correspond to almost identical spectra at O K-edge. This similar spectral behavior can be explained by areas with more or less single flakes deposited arbitrarily on top of each other. It seems that the thicker parts are not related to stacked layers of MXene flakes with strong interlayer interaction. Thus, the surface chemistry of few-layered Ti_3_C_2_T_x_ MXene is similar to single-layer MXene flakes, irrespective of the thickness. That may differ from multilayered Ti_3_C_2_T_x_ MXene that have not been delaminated.

**Figure S6** includes the TEY image or the same Ti_3_C_2_T_x_ MXene flakes. The signal-to-noise ratio of the TEY spectra is affected by the carbon deposition occuring during the characterization in vacuum (~10^-6^ mbar). Given the intraflake electron transport along MXene flakes, the area with the lowest TEY signal is a single flake, not well attached to the rest of the MXene sample.


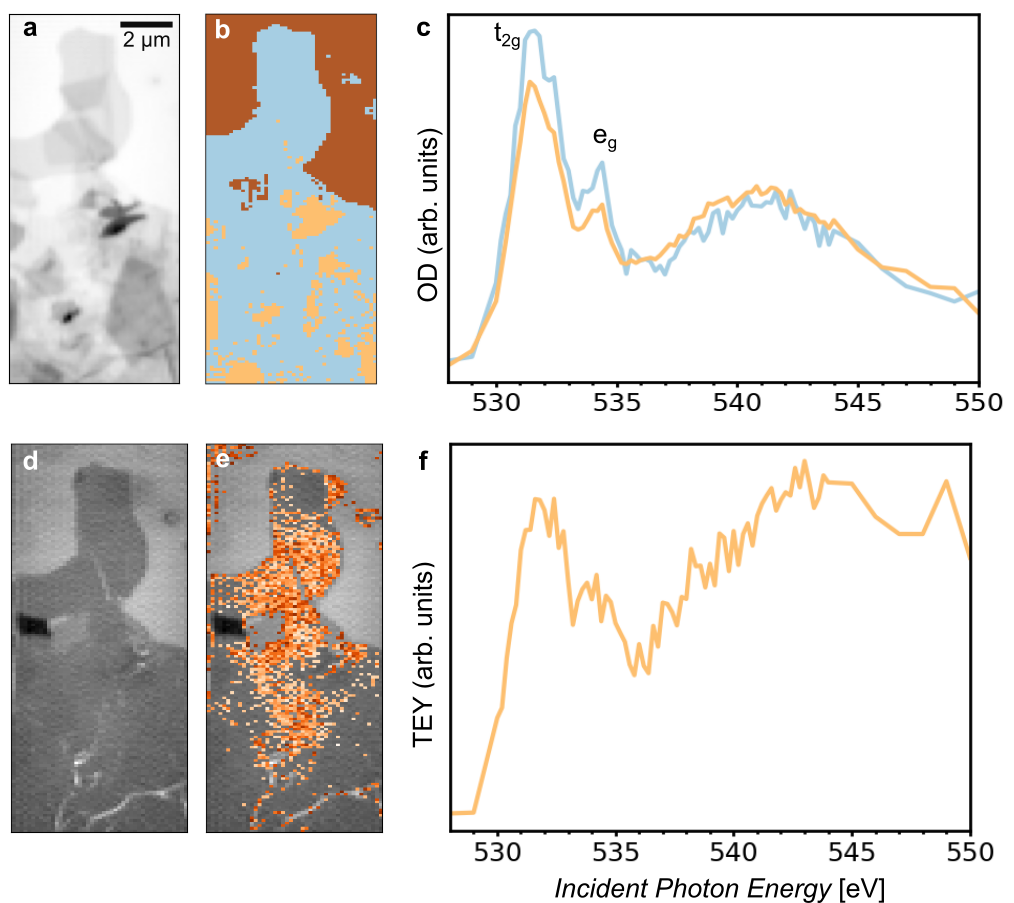


**Figure S6.** Transmission (**a**) and TEY (**d**) images, averaged over the energies at 528-550eV, for few-layered Ti_3_C_2_T_x_ MXene. The clustered transmission (**b**) and TEY image (**e**) with the corresponding transmission c and TEY spectra f.

##

## 5. Physical effects in transmission and TEY

Some physical effects in transmission and TEY may sometimes be observed as exemplified in the two SXM images in transmission and TEY shown in **Figure S7** taken at 450 eV (**Figure S7** **a** and **d**, respectively), and the other at the peak (458 eV) of maximum absorption (**Figure S7** **b** and **e**, respectively). By subtracting the pre-edge image from the image at the peak absorption maximum, a differential image that visually emphasizes the disparities between these two specific energy values can be obtained **Figure S7** **c** and **f** for transmission and TEY, respectively.

The monolayer flake (dotted violet) has a lower OD than the neighboring flake (dotted red) at the pre-edge but the differential image in transmission mode does not present any difference in absorption between these flakes. This means that the absorption between the two flakes is the same. It indicates that the non-conductive flake is most probably also a monolayer flake. However, the thickness in the area is increased due to chemical species not containing titanium.

The above assumption is confirmed by the measurements in TEY mode. The silicon nitride window is a conductive substrate, compared to the MXene sample that does not emit electrons at the pre-edge. Whereas the non-conductive flake has dramatically lower TEY signal than the monolayer flake at the maximum absorption peak, the differential image does not present much difference between the images. Hence, the non-conductive flake emits electrons comparable to the monolayer flake, if we eliminate the contribution of the substrate conductivity.

Finally, the green-dotted flake, laying on top of the flake (dotted with cyan), is darker in the initial transmission image, but cannot be separated from the bigger cyan-dotted flake in the differential image. This flake presents similar behavior with the non-conductive flake. However, it emits more electrons because it is not positioned directly to the substrate but on top of another flake.


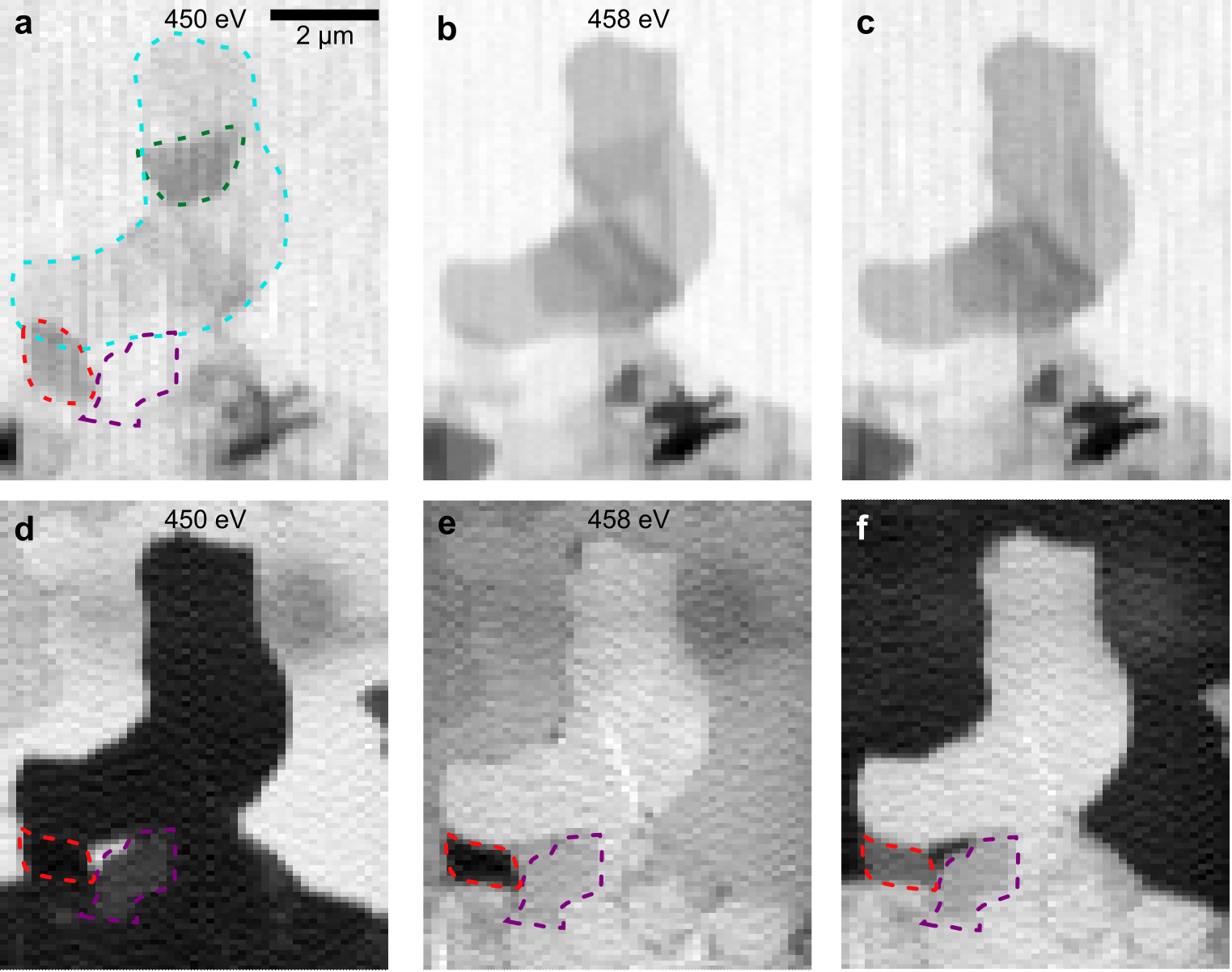


**Figure S7.** **Thickness effect**: SXM measurements for few-layered Ti_3_C_2_T_x_ MXene. **a** Transmission image at 450 eV (pre-edge region), **b** 458 eV (peak of maximum absorption) and **c** the difference between the two images. **d-f** The equivalent TEY images are presented.

Aside from the thickness effects in transmission, we also encounter carbon deposition, charging phenomenon and edge enhancement in TEY. In general, in transmission (TEY) measurements the counts in silicon nitride window without sample are increasing (decreasing) with increasing incident photon energy. For TEY, this is mainly explained by carbon deposition during scanning. However, this is not always the case as shown on SXM measurements for pristine multilayered Ti_3_C_2_T_x_ MXene partially covering both the silicon nitride window and the thicker (and conductive) silicon frame shown in **Figure S8**. The thick silicon frame does not allow transmission (**Figure S8 a**), unlike the silicon nitride window. The blue-colored MXene particle (Cluster 1) is thicker than the violet-colored (Cluster 2) in **Figure S8 b**, and thus the higher OD signal (**Figure S8 c**), attributed to higher thickness rather than higher absorption.

**Figure S8 d-e** presents the TEY measurements for the same MXene particle, where the counts on the silicon frame (Cluster I) decrease with increasing incident photon energy **Figure S8 f**. Surprisingly, the counts on the silicon nitride window (Cluster II) increase with increasing incident photon energy. This uncommon effect can be explained by the charge transfer during scanning. Fortunately, TEY spectrum is acquired for the MXene particle on top of both silicon nitride window and silicon frame. MXene on top of the silicon nitride window (Cluster III and IV) has lower counts the one on top of the more conductive silicon frame (Cluster V and VI). Clusters IV and VI present higher TEY signal than the Clusters III and V areas, respectively, as a result of edge enhancement phenomenon. We noticed that the TEY signal of Cluster V and VI not only decrease linearly at the region 450-454 eV, but after 454 eV an additional decrease of the signal is encountered due to the charging phenomenon. The decrease due to the charging phenomenon is also encountered in the substrate. This can be explained by the fact that the electrons from the charging MXene are transferred during scanning to the substrate, leading to its subsequent charging.


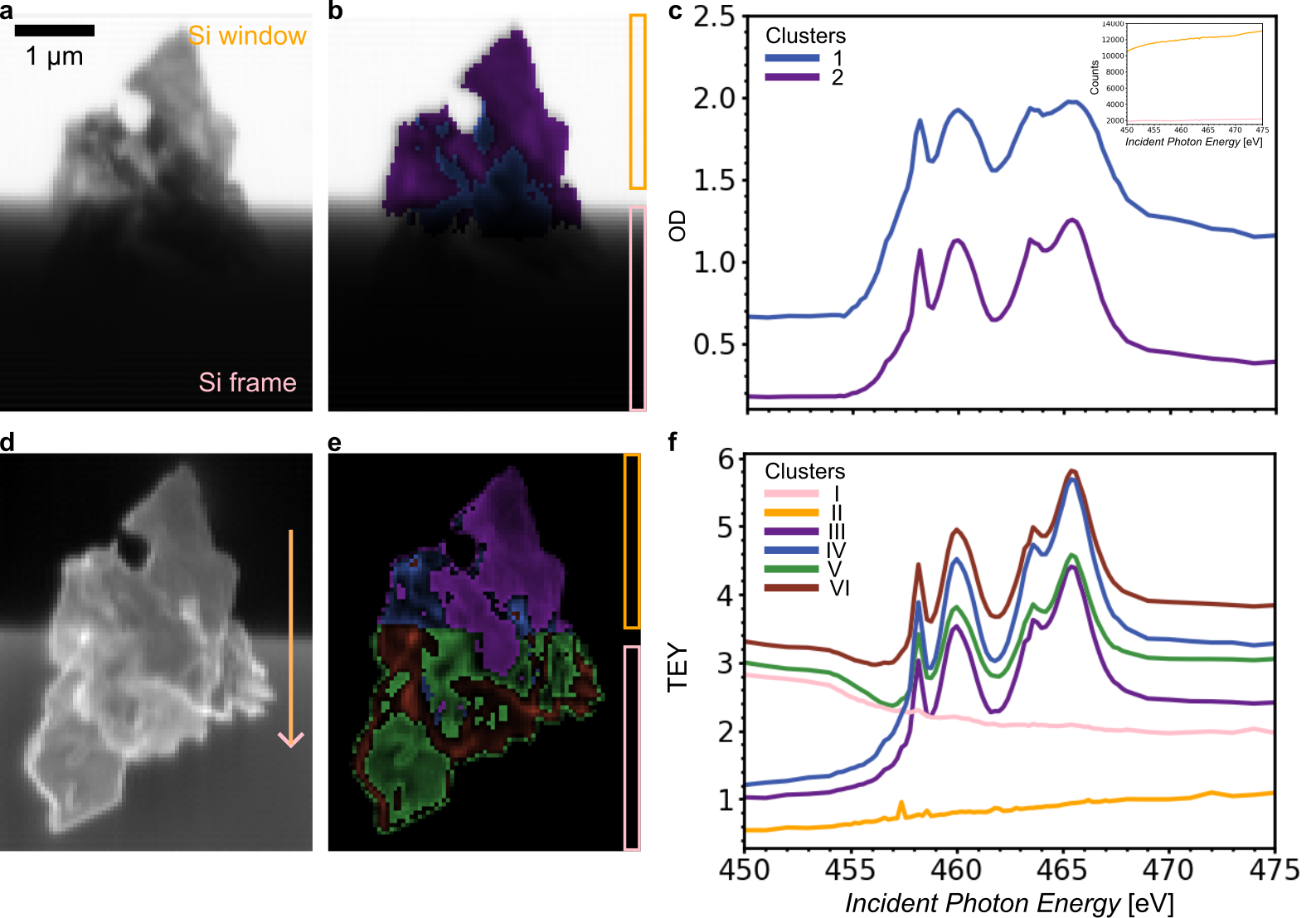


**Figure S8.** **TEY phenomena**: SXM measurements for pristine multilayered Ti_3_C_2_T_x_ MXene. **a** Transmission image, averaged at Ti L-edge, **b** corresponding clustered image, and **c** OD spectra with the I_o_ values showed in the inset. **d** TEY image, averaged at Ti L-edge, with arrow indicating the scanning direction, **e** corresponding clustered image, and **f** uncorrected TEY spectra.

## Electrochemically cycled MS- etched Ti_3_C_2_T_x_ MXene

Sonicated molten salt-shielded (MS^3^)-etched MXene is cycled 2 times in a classical Lithium-ion battery, and the cyclic voltagramms are recorded **(Figure S9**). The parts of the battery are transferred to the scanning X-ray microscope for spectromicroscopy, which require a short exposure to ambient air.


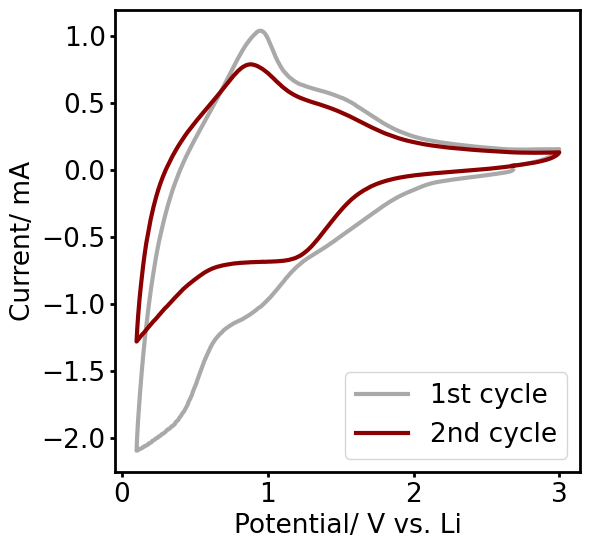


**Figure S9.** Cyclic voltammograms of sonicated molten salt-shielded (MS^3^)- etched MXene, cycled in a classical Li-ion battery (2 cycles from 3 V to 0 V).

In our study, we present images processed using Independent Component Analysis (ICA) for dimensionality reduction, followed by clustering through the Gaussian Mixture Model (GMM). **Figure S10 a** and **c** display the clustered transmission and TEY images, respectively, at O K-edge. The associated spectra are depicted in **Figure S10** **b** and **d**. In **Figure S10 b**, Clusters 1, 2 and 3 correspond to regions of cycled MXene with decreasing thickness. Clusters 4, 5 and 6 correspond to separator, carbonate species, and transparent silicon nitride window. In **Figure S10 d**, Clusters I and II correspond to regions of carbonate- electrolyte species with decreasing thickness. Clusters III and IV correspond to regions of carbonate- electrolyte species mixed with cycled MXene with decreasing thickness.


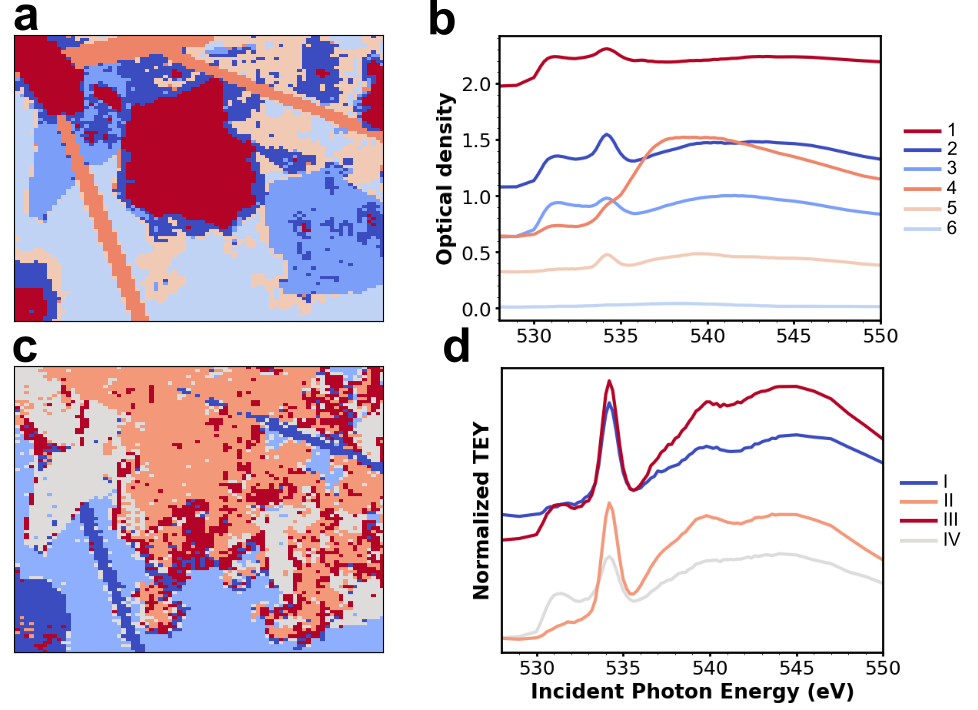


**Figure S10.** Clustered transmission and TEY image (**a** and **c**) and corresponding spectra (**b** and **d**) at O K-edge. The dimensionality reduction method is ICA and the clustering technique GMM.

**Figure S11** presents the proper way of defining the background subtraction. Equation S1 mentions that OD is estimated by OD= -ln(I/I_o_) and TEY signal is divided by I_o_, where I_o_ is the incident photon energy. For transmission datasets, I_o_ is estimated by averaging the spectra of the pixels that correspond to the silicon nitride window without material. However, the same technique cannot always be applied to TEY datasets for two reasons: (i) there are emission phenomena related to the silicon nitride window, and not to the MXene sample and (ii) the TEY signal from MXene areas does not include contribution from the window, given that TEY is a surface-sensitive technique. **Figure S11** **a** presents the uncorrected clustered TEY spectra, whereas **Figure S11** **b** presents the clustered TEY spectra, divided by the average spectrum of a manually selected area in the clean silicon nitride window. However, this results to noisy corrected spectra. To avoid that we need to include more pixels, whose spectra are averaged to estimate the proper I_o_ value. **Figure S11** **c** presents the clustered TEY spectra, divided by the average spectrum of a clustered area, corresponding to clean window.


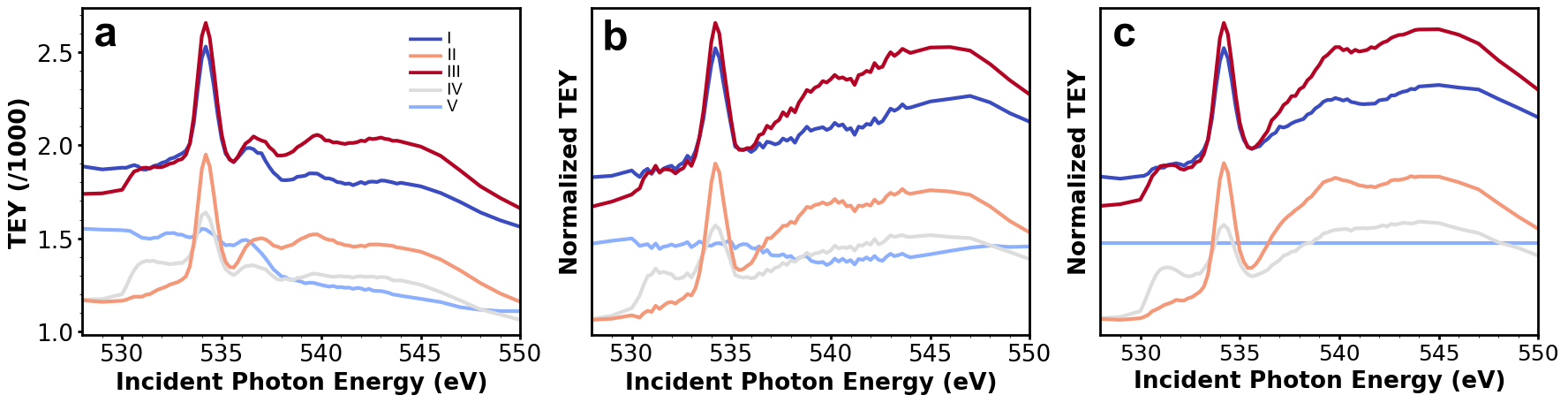


**Figure S11.** Clustered TEY spectra **a** without background correction, **b** by dividing with the average spectrum of a manually selected area, and **c** by dividing with the average spectrum of a clustered area. Spectra are derived from clustering the pixels of TEY images, by ICA and GMM.

**Figure S12** presents the spectrum of electrolyte residues at O K-edge, without the contribution of MXene. We can observe 4 discreet peaks at 530.6 eV, 534.2 eV assigned to CO_3_^2-^ species, 536.6 eV, 539.8 eV and a broad peak between 544-545 eV. The existence of a small peak at 530.6 eV can be tentatively assigned oxygen holes from defects in the degraded solvent molecules^[3]^ or Li_2_O_2_ that is common component of SEI layer ^[4]^. The rest of the peaks are attributed to electrolyte residues on top of the MXene.


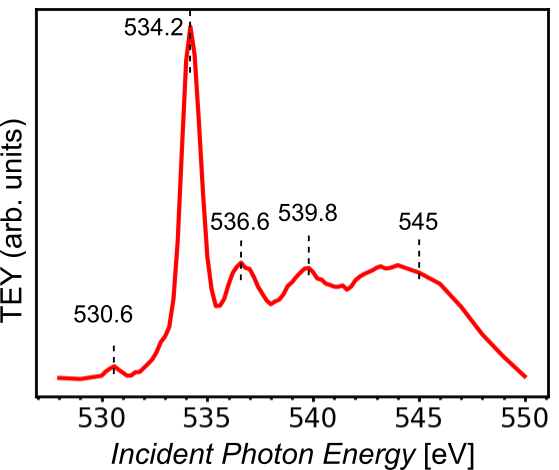


**Figure S12.** TEY-XAS at O K-edge of electrolyte residues.

**Figure S13** shows the fitted OD and TEY XA spectra of Figure 5 (c) and (g), respectively. The curve fitting analysis involves background subtraction with a background function (arc tangent) to remove the excitation of core electrons to the continuum or quasi-continuum states. Peak fitting is performed with Gaussian functions.

**Figure S13 a** are the fitted OD spectra at O K-edge, which represent the carbonates (red), pristine MXene (blue) and cycled MXene (green). Besides the components A_2_ (534.2 ± 0.2 eV), A_3_ (536.6 ± 0.2 eV), A_4_ (539.8 ± 0.2 eV) and A_6_ (545 ± 1 eV) that belong to the carbonate species, we notice two additional peaks at 531.4 eV (noted as A_1_) and at about 533.6 eV, overlapping with A_2_ component. These additional peaks belong to the molecular orbitals 2t_2g_ (Ti 3d+O 2p*π*) and 3e_g_ (Ti 3d+O 2p*σ*) of Ti_3_C_2_T_x_ MXene. Besides the t_2g_ and e_g_ peak, two more orbitals tentatively attributed to 3*a_1g_* (Ti 4s+O 2p*σ*) and 4*t_1u_* (Ti 4p+O 2p*π*) can be seen in energies above 535 eV for both pristine and cycled MXene. Depending on the oxidation state of MXene, these orbitals are fully or partially occupied. The main difference between pristine and cycled MXene is an additional peak between *t_2g_* and *e_g_* peak. The cyan-coded peak (532.2 eV) of cycled MXene is tentatively attributed to interaction of O-termination groups with Li cations during cycling.

**Figure S13 b** presents the TEY spectrum for the same area, previously attributed to cycled MXene. However, the corresponding TEY spectrum resembles the one for the carbonates. This is explained by the surface-sensitivity of TEY measurements, indicating that electrolyte residues and carbonate species lay on top of the cycled MXene. The majority of the sample’s top layer is described by the red TEY spectrum, which presents the characteristic components of the carbonates with only a weak signal of oxygen bond to titanium, highlighted with an arrow.

Overall, MXene’s main structure is preserved during cycling. OD spectra reveal bonding of lithium cations with MXene’s bulk for cycled MXene electrode. TEY spectra indicate that the carbonate species lay on top of the MXene.


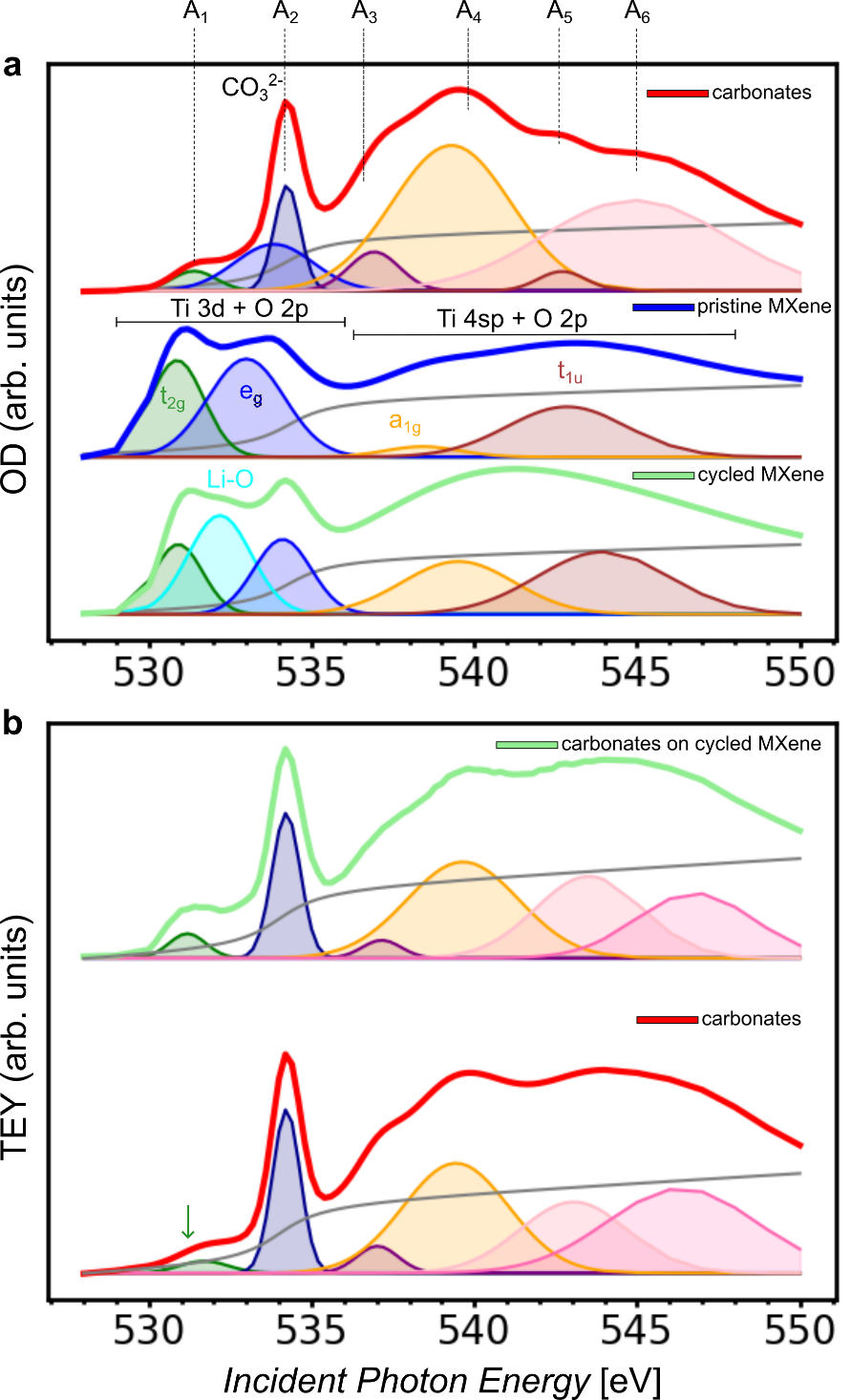


**Figure S13: Fitted spectra at O K-edge for cycled MXene in LP30 electrolyte**. (a) OD spectra at O K-edge, for pristine MXene (blue), cycled MXene (green) with signs of Li-O bond (cyan peak) and carbonates interacted with MXene (red). (b) Corresponding TEY spectra for carbonates with trace of oxygen bond to titanium.

## X-ray Diffraction patterns of MS- and HF-Ti_3_C_2_T*_x_* MXenes

**Figure S14** show that peaks of the MAX phases disappear from the XRD patterns in MXene, while the (00*l*) peaks shift to lower 2θ angles after either MS- or HF-etching, indicating the successful synthesis of both MXenes. In the case of HF-etched MXene, we include the XRD pattern of few-layered MXene with LiCl solution, indicating successful delamination.


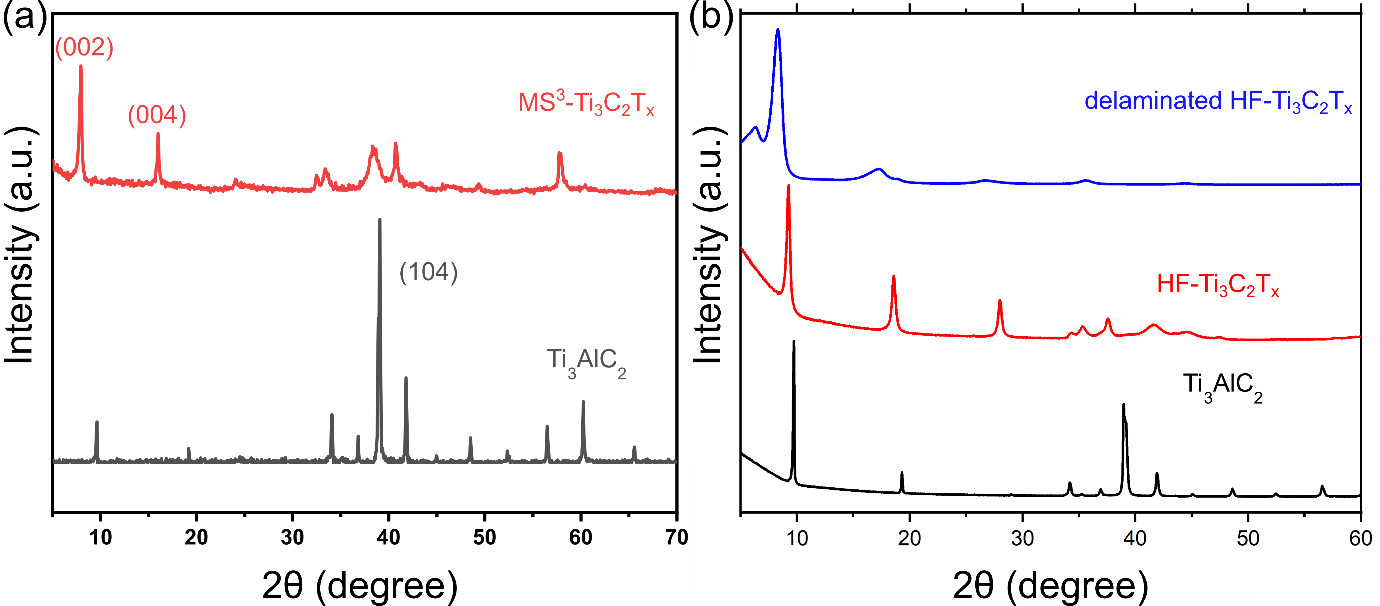


**Figure S14.** XRD patterns for (a) MS^3^-Ti_3_C_2_T_x_ MXene and its MAX precursor and (b) multilayered and few-layered HF-Ti_3_C_2_T_x_ MXene and their MAX precursor. The clear observation of the (002) and (004) peaks related to MXenes demonstrate the proper etching of the MAX phase for both methods.

## References

[1] [F. M. F. de Groot, J. C. Fuggle, B. T. Thole, G. A. Sawatzky, *Phys. Rev. B* **1990**, *41*, 928](https://doi.org/10.1103/PhysRevB.41.928).

[2] [K. W. Knehr, C. Eng, Y. Karen Chen-Wiegart, S. F. Zaccarine, M. Shviro, J. Nelson Weker, S. Spence, W.-K. Lee, F. Lin, X. Xiao, *Nanotechnology* **2021**, *32*, 442003](https://doi.org/10.1088/1361-6528/AC17FF).

[3] [T. Mizokawa, Y. Wakisaka, T. Sudayama, C. Iwai, K. Miyoshi, J. Takeuchi, H. Wadati, D. G. Hawthorn, T. Z. Regier, G. A. Sawatzky, *Physical Review Letters* **2013**, *111*, 056404](https://doi.org/10.1103/PHYSREVLETT.111.056404/FIGURES/4/MEDIUM)

[4] Qiao, R., Chuang, Y. D., Yan, S., & Yang, W. Soft X-Ray Irradiation Effects of Li2O2, Li2CO3 and Li2O Revealed by Absorption Spectroscopy. *PLOS ONE*, *7*(11), e49182, 2012
